# Phase II trial of fulvestrant plus enzalutamide in ER+/HER2− advanced breast cancer

**DOI:** 10.1038/s41523-023-00544-z

**Published:** 2023-05-20

**Authors:** Anthony D. Elias, Nicole S. Spoelstra, Alyse W. Staley, Sharon Sams, Lyndsey S. Crump, Gregory A. Vidal, Virginia F. Borges, Peter Kabos, Jennifer R. Diamond, Elena Shagisultanova, Anosheh Afghahi, Jose Mayordomo, Tessa McSpadden, Gloria Crawford, Angelo D’Alessandro, Kathryn L. Zolman, Adrie van Bokhoven, Yonghua Zhuang, Rosa I. Gallagher, Julia D. Wulfkuhle, Emanuel F. Petricoin III, Dexiang Gao, Jennifer K. Richer

**Affiliations:** 1 Division of Medical Oncology, Department of Medicine, University of Colorado Anschutz Medical Campus, Aurora, CO USA; 2 Department of Pathology, University of Colorado Anschutz Medical Campus, Aurora, CO USA; 3 Department of Pediatrics, University of Colorado Anschutz Medical Campus, Aurora, CO USA; 4 West Cancer Center and Research Institute and Dept of Medicine, University of Tennessee Health Sciences Center, Germantown, TN USA; 5 University of Colorado Cancer Center, Oncology Clinical Research Support Team, Anschutz Medical Campus, Aurora, CO USA; 6 University of Colorado Cancer Center, Cancer Clinical Trials Office, Anschutz Medical Campus, Aurora, CO USA; 7 Department of Biochemistry and Molecular Genetics, University of Colorado Anschutz Medical Campus, Aurora, CO USA; 8 Center for Applied Proteomics and Molecular Medicine, George Mason University, Manassas, VA USA

**Keywords:** Breast cancer, Breast cancer

## Abstract

This clinical trial combined fulvestrant with the anti-androgen enzalutamide in women with metastatic ER+/HER2− breast cancer (BC). Eligible patients were women with ECOG 0–2, ER+/HER2− measurable or evaluable metastatic BC. Prior fulvestrant was allowed. Fulvestrant was administered at 500 mg IM on days 1, 15, 29, and every 4 weeks thereafter. Enzalutamide was given at 160 mg po daily. Fresh tumor biopsies were required at study entry and after 4 weeks of treatment. The primary efficacy endpoint of the trial was the clinical benefit rate at 24 weeks (CBR24). The median age was 61 years (46–87); PS 1 (0–1); median of 4 prior non-hormonal and 3 prior hormonal therapies for metastatic disease. Twelve had prior fulvestrant, and 91% had visceral disease. CBR24 was 25% (7/28 evaluable). Median progression-free survival (PFS) was 8 weeks (95% CI: 2–52). Adverse events were as expected for hormonal therapy. *S*ignificant (*p* < 0.1) univariate relationships existed between PFS and ER%, AR%, and *PIK3CA* and/or *PTEN* mutations. Baseline levels of phospho-proteins in the mTOR pathway were more highly expressed in biopsies of patients with shorter PFS. Fulvestrant plus enzalutamide had manageable side effects. The primary endpoint of CBR24 was 25% in heavily pretreated metastatic ER+/HER2− BC. Short PFS was associated with activation of the mTOR pathway, and *PIK3CA* and/or *PTEN* mutations were associated with an increased hazard of progression. Thus, a combination of fulvestrant or other SERD plus AKT/PI3K/mTOR inhibitor with or without AR inhibition warrants investigation in second-line endocrine therapy of metastatic ER+ BC.

## Introduction

Breast cancer (BC) is a genetically heterogeneous and biologically diverse disease. We currently subdivide BC by estrogen receptor alpha (ER), progesterone receptor (PR), and human epidermal growth factor receptor (HER2/neu) status, in part because these markers represent important predictive biomarkers that guide treatment with discernible survival benefits. Endocrine therapies, such as tamoxifen, fulvestrant, and aromatase inhibitors (AI), target ER directly or the production of estrogen and play a critical role in the treatment of patients with ER+ disease^1^. Androgen receptors (AR) are expressed in most BC, and AR positivity (nuclear staining by immunohistochemistry (IHC) in 10% or more of cells) was observed in 77% of 3093 invasive breast tumors of all subtypes, including 91% of ER+ BC^2^. The predominately nuclear localization of AR by IHC^2^ indicates that it is in the liganded state. In primary ER+ BC compared to patient-matched metastatic disease, AR is commonly maintained in metastases, while ER often decreases^3^.

The functional role of AR in ER+ BC remains controversial^4^, with confusion arising from the fact that when estradiol is present, androgens decrease ER-mediated proliferation in cell lines and xenograft models^5^. In contrast, in the absence of estrogen, androgens stimulate proliferation, and AR is associated with resistance to tamoxifen and aromatase inhibitors (AI) in ER+ BC^3,6–10^. Tumors that respond to tamoxifen express similar percent cells positive for ER and AR protein, as does adjacent uninvolved epithelium^10^. However, tamoxifen-resistant breast tumors have a high ratio of percent cells positive for nuclear AR versus ER. A ratio of AR:ER ≥ 2.0 in primary tumors is associated with an over four-fold increased risk for failure while on adjuvant tamoxifen and overall disease-free survival, with an independent effect on risk for relapse beyond ER positivity alone^9^. Similar findings have been reported for patients treated with adjuvant AI therapy^11–13^.

While AIs effectively block the conversion of androgens to estrogens to decrease ER-stimulated tumor growth, over time, circulating and intra-tumoral androgens can increase as an unintended consequence^14–17^, resulting in AR activation and resistance to AI therapy. In the absence of estradiol (E2) (as in post-menopausal women on AI therapy) dihydrotestosterone (DHT) (which cannot be aromatized to estrogen) increased proliferation of ER+ cell lines and patient-derived xenografts^3,9,18,19^. On the other hand, the selective androgen receptor modulator enobosarm decreased tumor growth in preclinical models^5^. A recent trial NCT02007512 using the AI exemestane, with or without enzalutamide, in patients with ER+ advanced/metastatic disease found that high levels of *AR* and low levels of *ESR1* were associated with the significantly greater benefit of enzalutamide^20^. These complexities emphasize the importance of clinical context and hormonal milieu when considering AR action in BC^21^. Clearly, AR does influence BC biology, and high AR relative to ER levels can serve as an independent predictor of response to anti-estrogen therapies, perhaps by identifying tumors poised to escape ER-directed therapies and switch to survival dependent on androgens and AR. Thus, we postulated that when ER+ BC becomes resistant to ER-targeting therapies, agents targeting AR may provide clinical benefits.

The use of the anti-androgen enzalutamide showed efficacy in women with TNBC^22^, and it is now under investigation in women with ER+ BC. An extended phase I trial of enzalutamide identified 160 mg/day as the recommended phase 2 dose, and the PK and safety profile in women was similar to that observed in men^23^. Enzalutamide is a potent CYP3A4 inducer and reduced the areas under the concentration-time curve (AUC) of anastrozole and exemestane by 80% and ~50%, respectively. However, when combined with fulvestrant, no significant PK interaction or new safety signals were found^24,25^. Preclinical modeling showed synergistic inhibitory effects of fulvestrant plus enzalutamide on tumor cell growth^3^. Therefore, we hypothesized that enzalutamide, combined with fulvestrant, would be effective in patients with ER+ AR+ metastatic BC resistant to traditional therapeutic strategies targeting ER or estrogen production (AI therapy). The primary objective of the current trial was to determine CBR24 of the combination of enzalutamide and fulvestrant in metastatic BC originally diagnosed as ER+ disease. The secondary objectives were to confirm the safety profile of the combination, response rate, and progression-free survival (PFS) at 24 weeks. Serial biopsies were obtained during pretreatment and at the end of the fourth week of treatment (hence referred to as week 5) to evaluate the effects of treatment on the tumor and its relationship to clinical outcomes.

## Results

### Demographics (32 eligible patients evaluable for toxicity)

Of the 32 eligible participants, 28 were evaluable for response (Table 1). The median age was 61 [46–87] years, and the median ECOG PS was 1 [0–2]. Patients were heavily pretreated with a median of 3 [1,9] prior hormonal agents and 4 [0,8] prior non-hormonal therapies. Twelve (37.5%) patients had prior fulvestrant, and 29 (90.6%) had visceral disease.

**Table 1:** Patient characteristics.

Characteristic	Number (percentage/range)
Consented	38 (6 screens fail)
Eligible and evaluable	32 (100.0%)
*Age at the time of consent (years)*	
Median (range)	61 (46, 87)
*ECOG PS*	
Median (range)	1 (0, 2)
Adjuvant chemotherapy	14 (43.8%)
Neoadjuvant chemotherapy	5 (15.6%)
Adjuvant endocrine therapy	20 (62.5%)
Chemotherapy for metastatic disease	15 (46.9%)
Endocrine therapy for metastatic disease	25 (78.1%)
Prior fulvestrant	12 (37.5%)
*Prior agents for advanced breast cancer*	
Median (range)	7 (2, 15)
* Hormonal*	
Median (range)	3 (1, 9)
* Non-hormonal*	
Median (range)	4 (0, 8)
Metastatic sites	
* ≥3 metastatic sites*	17 (53.1%)
Bone	24 (75.0%)
Visceral	29 (90.6%)
Evaluable or measurable disease	28 (87.5%)
AR ≥ 10% Positive (*N* = 27)	20 (74.1%)
ER ≥ 10% Positive (*N* = 27)	22 (81.5%)
PR ≥ 10% Positive (*N* = 27)	12 (44.4%)
AR:ER Ratio ≥ 2 (*N* = 24)	5 (20.8%)
*% Baseline IHC Ki67 (N* *=* *27)*	
Median (range)	50 (0, 95)
*% Week 5 IHC Ki67 (N* *=* *27)*	
Median (range)	40 (3, 95)
*ESR1* mutation	11 (34.4%)
*P53* (*N* = 29)	14 (48.3%)
*PTEN/PIK3CA (N* *=* *29)*	
* PIK3CA* and *PTEN*	3 (10.3%)
* PIK3CA* Only	8 (27.6%)
* PTEN* Only	3 (10.3%)
Neither	15 (51.7%)

*aBC* advanced breast cancer, *AR* androgen receptor, *ECOG PS* Eastern Cooperative Oncology Group Performance Status, *enza* enzalutamide, *ER+/PgR+* estrogen receptor–positive/progesterone receptor–positive, *HER2* human epidermal growth factor receptor 2.

Treatment-emergent adverse events (AEs) were consistent with what would be expected with hormonal therapy. Fatigue, nausea, and achiness were most common. Cognitive dysfunction described as “difficulty concentrating” was reported in 5 patients and resolved upon completion of treatment. AEs greater than 20% included fatigue (53.1%), nausea (50.0%), vomiting (28.1%), constipation (31.2%), anorexia (28.1%), headache (34.4%), achiness (43.8%), and tumor-associated pain (31.3%). Most AEs were low-grade. G3 toxicity was uncommon and, in most cases, was unrelated to protocol treatment. There were no G4 or G5 toxicities (Table 2).

**Table 2:** Adverse events.

AE	Any grade (*N* = 32)	G3 related (*N* = 32)
*Constitutional*		
*symptoms*		
Fatigue	17 (53.1%)	2 (6.2%)
Hot flashes	6 (18.8%)	0
Insomnia	6 (18.8%)	0
Anxiety	4 (12.5%)	0
*Gastrointestinal*		
Nausea	16 (50%)	0
Vomiting	9 (28.1%)	1 (3.1%)
Diarrhea	7 (21.9%)	0
Constipation	10 (31.2%)	0
Anorexia	9 (28.1%)	0
Dyspepsia	3 (9.4%)	0
*Neurologic*		
Cognitive disorder	5 (15.6%)	0
Lightheaded	6 (18.8%)	0
Headache	11 (34.4%)	0
*Miscellaneous*		
Achiness	14 (43.8%)	1 (3.1%)
Itch/Rash	3 (9.4%)	0
UTI	4 (12.5%)	1 (3.1%)
Hair Loss	1 (3.1%)	0
Tumor-associated pain (NR)	10 (31.3%)	0

Back pain (9) and hepatic pain (1) reclassified as tumor-associated pain (NR).

Flatulence, malaise, dry mouth, visual changes, stomach cramps, T wave flattening, breast swelling, nails peeling (one each, G1/2). No G4 or G5 toxicities.

### CBR and overall PFS (28 evaluable patients)

**Fig. 1:**
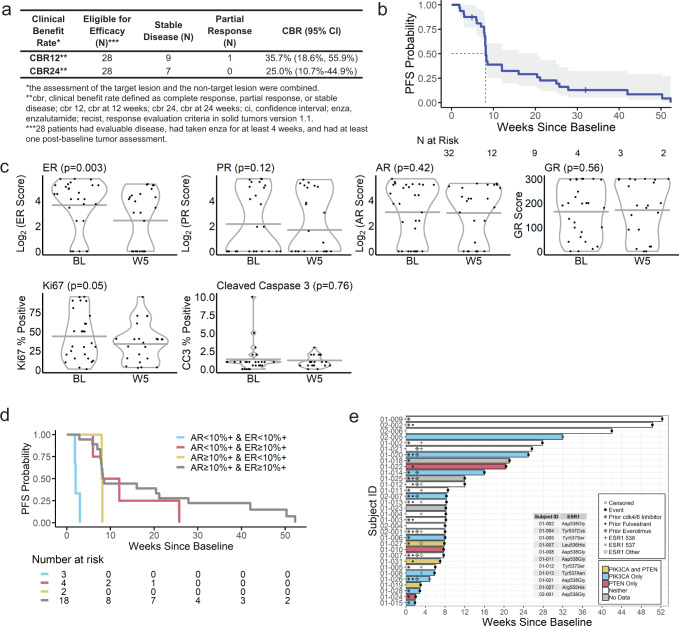
Efficacy of enzalutamide plus fulvestrant in advanced ER+/HER2− breast cancer patients. **a** Clinical benefit rate at 12 weeks (CBR12) and 24 weeks (CBR24). **b** Kaplan–Meier of progression-free survival (PFS) in all patients treated with enzalutamide plus fulvestrant. Censored times are marked with vertical dashes, and the median time to progression (8 weeks) is noted. **c** Violin plots of steroid hormone receptor IHC quantification ER, AR, PR, GR, as well as Ki67 and Cleaved Caspase 3 (CC3) IHC, are stratified by baseline (BL) and week 5 (W5) of treatment. Plots show the distribution of the observed values with the time-specific minimum and maximums as the lowest and highest gray horizontal lines, respectively. Corresponding *p*-values and black points indicate observed values as assessed with the Wald two-sided test. **d** Kaplan–Meier curve of overall survival probability in patients treated with enzalutamide plus fulvestrant stratified by AR and ER protein expression as determined by immunohistochemistry. **e** Swimmer plot representing each patient’s progression-free survival time in weeks. Censored end times are marked with open circles, and participants who experienced an event are marked with black squares. *PIK3CA* and *PTEN* are represented by color, and prior treatments and biomarkers are indicated with symbols.

At week 24, 7 (25.0%) (95% CI: 10.7–44.9) participants had stable disease, and no participants had a partial response (Fig. 1a). The median time to progression was 8 weeks (95% CI: 8–20) (Fig. 1b).

### Immunohistochemistry for hormone receptors and Ki67

The association between time and each IHC outcome was assessed with univariate linear mixed models. On average, ER Score was 63.3 (95% CI: 24.9–101.6; *p* = 0.003) units lower at week 5 than at baseline, and Ki67 was 10.9 (95% CI: 0.1–21.8; *p* = 0.05) percentage points lower at week 5 than at baseline. No other IHC variable, including PR and glucocorticoid receptor (GR), had a significant difference between baseline and week 5 of treatment (Fig. 1c).

### Demographic and clinical risk factors of progression: multivariate Cox proportional hazard survival analysis

Dichotomous AR and ER percent positive at baseline were included in the multivariate PFS model. Due to sample size restrictions, *PIK3CA*/*PTEN* and prior fulvestrant were excluded. The hazard of disease progression for participants with AR < 10% positive was 2.46 (95% CI: 0.97–6.22) times the hazard for participants with AR ≥ 10% positive after controlling for ER percent positive (*p* = 0.057). The hazard of disease progression for participants with ER < 10% positive was 4.69 (95% CI: 1.53–14.35) times the hazard for participants with ER ≥ 10% positive, after controlling for AR percent positive (*p* = 0.007) (Table 3, Fig. 1d).

**Table 3:** Univariate and multivariate analyses.

Time to disease progression			
	Hazard ratio	CI	*p*
*Univariate predictors*			
AR < 10% Positive (*N* = 27)	2.28	0.93–5.62	0.073
ER < 10% Positive (*N* = 27)	4.32	1.46–12.83	0.008
AR and/or ER < 10% Positive (*N* = 27)	2.35	0.99–5.58	0.052
PR < 10% Positive (*N* = 27)	0.73	0.32–1.67	0.452
*ESR1* mutation (*N* = 32)	1.47	0.68–3.19	0.329
Ki67 decreased^a^ (*N* = 21)	1.79	0.70–4.59	0.225
*PTEN* and/or *PIK3CA* (*N* = 29)	2.02	0.89–4.56	0.092
*Multivariate predictors (N* *=* *27)*			
AR < 10% Positive	2.46	0.97–6.22	0.057
ER < 10% Positive	4.69	1.53–14.35	0.007

Select univariate associations with progression are presented. The reference level for “AR and ER < 10% Positive” are participants with AR ≥ 10% positive and/or ER ≥ 10% positive. The reference level for “PTEN and/or PIK3CA” are patients with neither loss. All other univariate associations of interest were non-significant predictors of progression, with *p*-values greater than 0.2.

^a^Ki67 decreased refers to patients who had a decrease in Ki67 between baseline and week 5. The reference group is patients who had an increase or no change in Ki67 between baseline and week 5.

A Kaplan–Meier curve of PFS stratified by AR and ER protein by IHC suggested that those with biopsies with <10% positive cells for both ER and AR had the shortest PFS, and those with ≥10% of both receptors received the longest PFS, albeit sample size was too limited to perform statistical tests (Fig. 1d).

### Demographic and clinical risk factors of progression: Univariate survival analysis

*S*ignificant (*p* < 0.2) univariate relationships were observed between continuous PFS and the following variables: ER and AR percent positivity by IHC and *PIK3CA and/or PTEN* mutations. Specifically, the average hazard for progression was 4.32 (95% CI: 1.46–12.83) times higher for participants with ER < 10% positive compared to participants with ER ≥ 10% positive (*p* = 0.008). This association remained significant when ER was considered continuous (*p* = 0.024). Additionally, the average hazard of progression was 2.28 (95% CI: 0.93–5.62) times higher for participants with AR < 10% positive compared to participants with AR ≥ 10% positive (*p* = 0.073). The hazard of disease progression for participants with AR and/or ER < 10% positive was 2.35 (95% CI: 0.99–5.58; *p* = 0.052) times the hazard for participants with AR and/or ER ≥ 10% positive. Finally, the hazard of disease progression for patients with *PTEN* and/or *PIK3CA* mutated tumor biopsies was 2.02 (95% CI: 0.89–4.56; *p* = 0.092) times the hazard for participants with neither molecular event. This is illustrated in a swimmer plot of PFS, prior fulvestrant, and prior everolimus with activating mutations in the *PIK3CA* or potential loss of function *PTEN* mutations, as well as *ESR1* mutations, indicated (Fig. 1e). No other factors of interest, including *ESR1* mutations, prior exposure to fulvestrant (38%), everolimus (28%), or cdk4/6 inhibitors (83%) had significant univariate associations with PFS (Table 3, Fig. 1e).

### Reverse phase protein array demonstrates activation of the mTOR pathway in tumors from patients in the short PFS group

**Fig. 2:**
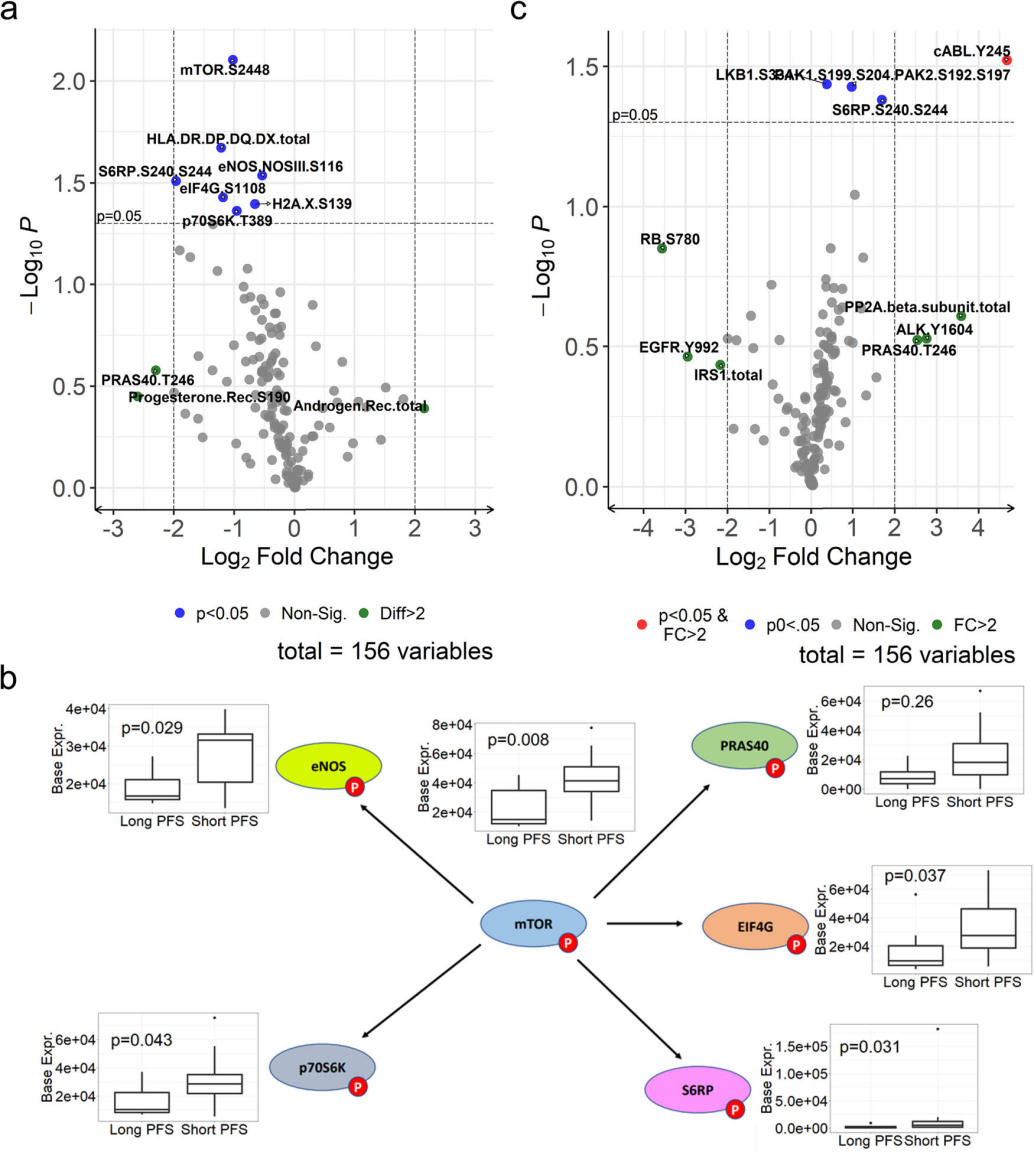
RPPA analysis of frozen core biopsies from tumors at baseline and following enzalutamide plus fulvestrant. **a** Volcano plot of differences in baseline detection across the Long and Short PFS groups. **b** Differentially expressed phospho-proteins in the mTOR signaling pathway in Long PFS versus Short PFS at baseline. The lower and upper bounds of the boxes represent the first (Q1) and third (Q3) quartiles, respectively, which cover the interquartile range (IQR). The horizontal line within each box represents the median. The whiskers extending vertically out from the boxes are computed as Q1 − 1.5*IQR and Q3 + 1.5*IQR. Outliers are data points outside of this range and are indicated with dots. **c** Volcano plot of differences in fold change detection across Long and Short PFS groups. All statistical analyses were performed with the Bayes log2 two-sided moderated *t*-test.

When comparing phosphorylated proteins in the “Short PFS” defined in all RPPA analyses as PFS ≤ 60 days (*n* = 20) to those who experienced “Long PFS” defined as PFS > 24 weeks (168 days) (*n* = 7), the mTOR pathway had higher baseline expression levels in the tumors of those with Short PFS. According to robust moderated *t*-tests, baseline mTOR S2448 (*p* = 0.008), eNOS/NOSIII S116 (*p* = 0.029), S6RP S240/S244 (*p* = 0.031), eIF4G S118 (*p* = 0.037), and p7056K T389 (*p* = 0.043) were significantly higher in the Short PFS group compared to the Long PFS group (Fig. 2a and Supplementary Table 1). Similar results were obtained when Short PFS was defined as <24 weeks (*n* = 25). This suggests mTOR activation in the pre-treatment tumor biopsies from patients with a short time to progression (Fig. 2b). However, the differences in the baseline abundance of each mTOR protein were not significantly different between tumors with *PTEN*/*PIK3CA* mutants versus those without these mutations and were also not associated with prior everolimus.

We also assessed the RPPA data for phospho-proteins that changed pre- versus post-enzalutamide plus fulvestrant in patients with Short versus Long PFS. Proteins differentially altered by treatment in the Short PFS group compared to the Long PFS group included phosphorylated forms of cABL Y245, LKB1 S334, PAK1/PAK2 S199, S204/S192, S197 and S6RP S235, S236, and RB S780, respectively (Fig. 2c and Supplementary Table 2).

### Phospho-proteins that correlate with ER or AR at baseline and with the change after treatment

There were 74 (47.1%) proteins that had significant baseline associations with total ER in the RPPA data (Supplementary Table 3a), 72 (97.3%) of which were positively correlated, meaning that patients with lower ER tended to have lower values of correlated proteins and patients with higher ER tended to have higher values of correlated proteins. Additionally, 86 (54.8%) of proteins had a significant association with the change in ER; 85 (98.8%) of which were positively correlated (Supplementary Table 3b). Of note, total ER positively correlated with ERpS118, PRpS190, total AR, ARpS81, as well as total Cyclin D1 and many other interesting phospho-proteins. Many of the same proteins changed along with ER following treatment.

Similarly, 48 (30.6%) proteins had significant baseline associations with AR, and 80 (51.0%) proteins had significant fold change associations with AR. Respectively, 48 (100%) and 79 (98.8%) were positively correlated (Supplementary Table 4a, b). pARS81 correlated with total AR at baseline, as did total Cyclin D1, total ER, and total HER2. These proteins also changed with AR following treatment.

Proteins that correlated with ER and AR were enriched for several pathways (Supplementary Tables 5a, b and 6a, b). Response to estradiol and epithelial cell proliferation corresponded with baseline ER levels and the change in ER with treatment (Supplementary Table 5a, b). Proteins correlated with baseline AR corresponded to hair follicle development, negative regulation of heart valve morphogenesis and mesenchyme morphogenesis, and negative regulation of transcription and translation (Supplementary Table 6a), and some of the same pathways and embryonic development changed with AR with treatment as well (Supplementary Table 6b).

### Plasma metabolomics analyses

**Fig. 3:**
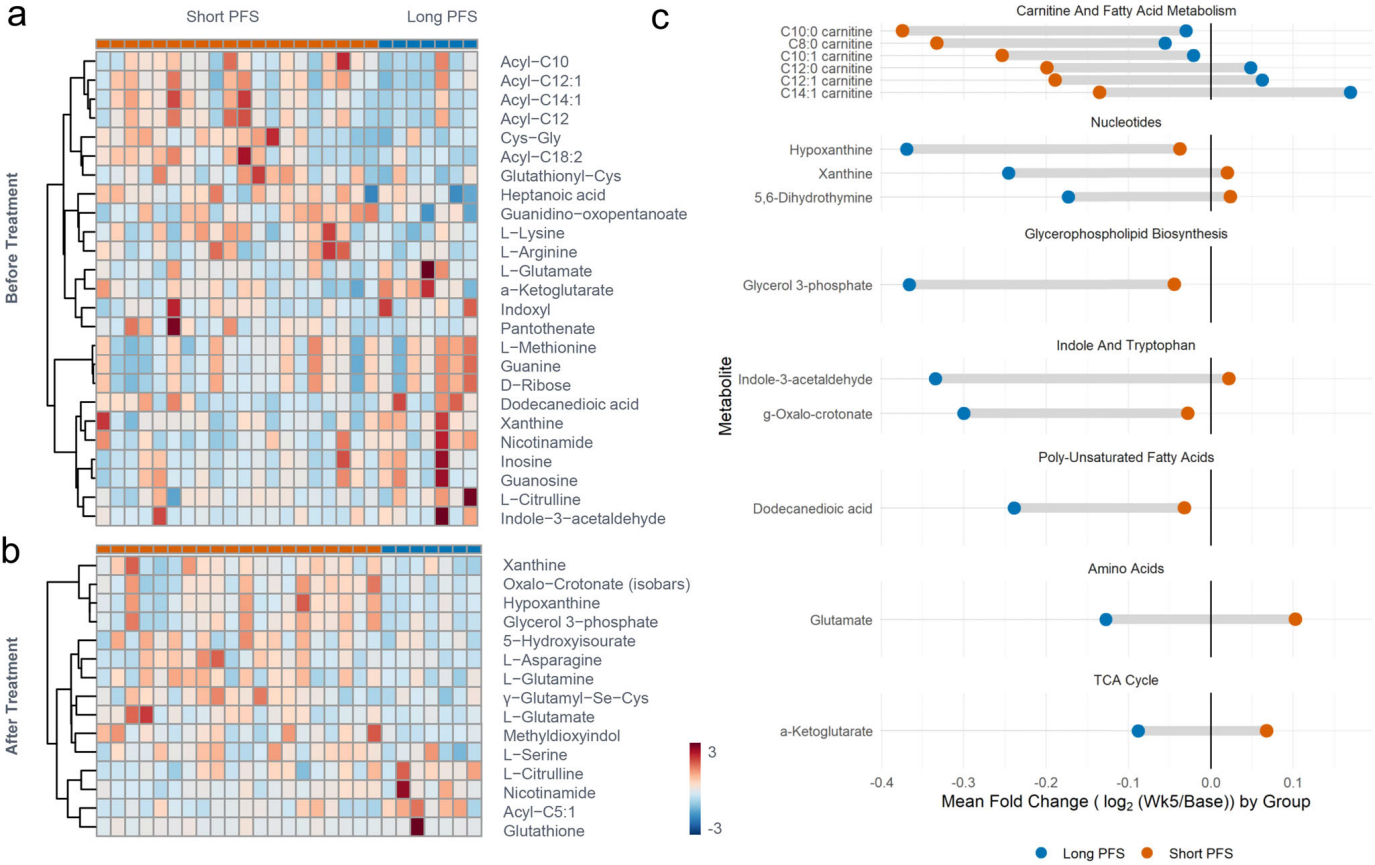
Analysis of plasma metabolomics performed via UHPLC-MS at baseline and after treatment. Metabolites with significant differences between Long and Short PFS are presented in heatmaps **a** at baseline and **b** after treatment. **c** The mean fold changes with treatment are presented for metabolites with significant differences between the Long and Short PFS groups.

Metabolomics analyses of patient plasma revealed baseline and post-treatment differences in acyl-carnitines, amino acid, and purine metabolism in patients with Short versus Long PFS. Given the role of mTOR as a master sensor and regulator of metabolism^19,26,27^, compared to Long PFS patients, plasma from patients with Short PFS had higher levels of several acyl-carnitines (C10, 12, 12:1, 14:1, 18:2), but a lower level of amino acids (l-arginine, citrulline, glutamate, lysine, methionine) and purine breakdown and deamination products (inosine, xanthine, guanosine) at baseline (Fig. 3a). Following treatment, purine oxidation products (hypoxanthine, xanthine, 5-hydroxyiusourate) and amino acids (asparagine, glutamine, glutamate) were higher in the Short PFS group (Fig. 3b). However, serine, citrulline and the main soluble intracellular antioxidant tripeptideglutathione, were higher in plasma from Long PFS patients (Fig. 3b). This is relevant considering the role of mTOR in amino acid sensing—especially serine^28^. Increased consumption of fatty acids and preservation of plasma acyl-carnitine levels were observed following treatment in Long PFS patients. Based on these results, both liver functions, transamination (glutamate, alpha-glutamate), and glutathione metabolism are elevated in the plasma of the Short PFS group.

## Discussion

The current phase II clinical trial investigated the combination of fulvestrant with enzalutamide in a heavily pretreated population of women with metastatic ER+/HER2− BC. The regimen achieved CBR24 in 25% of the patients, with generally low-grade toxicity with no new safety signals. It is notable that 29% (2 of 7) patients in the Long PFS (PFS > 24 weeks) group had prior exposure to fulvestrant. Analyses of tumor biopsies demonstrated that patients with ≥10% positive cells for ER or AR by IHC had longer PFS than patients with ER and AR < 10% positive. Metastatic disease evolves under the selective pressure of treatments and can lose expression of ER^29,30^, and this was observed in 13% of the patients in this study. In primary tumors versus patient-matched metastases, we previously found that nuclear AR was often maintained even when ER was reduced or absent^3^. These data emphasize the need to consider fresh biopsy to confirm the presence of the therapeutic targets of interest.

Patients with Short PFS (PFS ≤ 60 days) had increased mTOR pathway activation in the pre-treatment biopsies compared to patients with PFS > 24 weeks. Interestingly, these findings were supported by plasma metabolomics data, showing significantly lower levels of multiple amino acids in patients with Short PFS (<24 weeks) compared to Long PFS (>24 weeks)—an observation that would be consistent with mTOR activation via sensing of systemic amino acid depletion^31^. In addition, patients with Long PFS are characterized by preservation of circulating levels of lipid oxidation markers (acyl-carnitines) compared to patients with Short PFS, suggestive of preserved mitochondrial function^32^, in the former group following treatment with fulvestrant with enzalutamide. This suggests potential utility for adding an mTOR inhibitor such as everolimus to enzalutamide, particularly for patients with activated mTOR or PIK3CA/PTEN mutations, to potentially enhance response rate as has been suggested previously by preclinical and clinical studies^19,33^*. PIK3CA* and *PTEN* mutations are frequent in metastatic ER+ BC^34,35^. In this study, we find that *PIK3CA* activating and or *PTEN* inactivating mutations were significantly associated with a greater hazard of PFS.

Recently, a randomized phase II trial comparing the aromatase inhibitor exemestane alone (25 mg daily) to exemestane (50 mg daily) plus enzalutamide was completed^20^. Overall, 247 patients with ER+ BC were randomized into two cohorts (one with no prior endocrine therapy for metastatic disease and the other with one prior endocrine therapy in that setting). In this study, only the cohort without prior endocrine therapy had a numerical advantage in PFS for the addition of enzalutamide in patients with high *AR* (above the median), particularly in combination with low (below median) levels of *ESR1* mRNA in their primary tumors. These patients had a reduced risk of progression or death compared to the control arm [HR, 0.24 (95% CI: 0.10–0.60); *p* = 0.001, with the median PFS extended by 10.2 months (from 3.8 in the control arm to 14.0 months in the enzalutamide arm)^20^. These data may suggest that primary tumors with higher-than-average *AR* but lower-than-average *ESR1* are poised to become resistant to AI therapy and may benefit from anti-androgen therapy. This is in line with the high AR to ER percent cells positive data summarized in the introduction^36^. Interestingly, enobosarm, a selective androgen receptor modulator (SARM) with both agonist and antagonist activity depending on tissue type, showed activity in preclinical^5,37^ and clinical studies^38^. In women with endocrine-sensitive ER+ BC on adjuvant endocrine therapy (ET) for at least 3 years or who responded to the most recent ET for metastatic disease for at least 6 months, CBR24 was 32% for those treated with enobosarm (9 mg daily), and 29% for those in the 18 mg cohort. Responses were observed in patients with AR+ BC (>10% nuclear AR staining)^38^. Another recent trial examined the efficacy and safety of enzalutamide with trastuzumab in patients with HER2+/AR+ locally advanced or metastatic BC in a single-arm phase II study in heavily pretreated patients with advanced HER2+ AR+ BC and observed a 24% CBR24^39^.

A major strength of NCT02953860 includes paired tumor biopsies pre-treatment and on-treatment to assess mutations, phospho-protein expression, and associations with PFS. We find that 7 (25%) of patients reached CBR24, including 2 who were progressing on prior fulvestrant, suggesting possible benefit for this subset. The study lends insight into the pathways that might be of importance regarding resistance, such as mTOR activation. Other than ER ≥ 10%, AR ≥ 10%, and the absence of *PTEN* or *PIK3CA* mutations, there were no clear indicators of response. Limitations include a heavily pretreated population with substantial heterogeneity of prior treatment. The sample size is limited; thus, all correlations are by nature exploratory. Future research must be conducted to evaluate the reproducibility of the results. Regarding the interpretation of the plasma metabolomics, we acknowledge that system-wide metabolomics analyses cannot distinguish between tumor versus organ response to drugs; however, the differences between the Short and Long PFS groups are intriguing. These analyses were performed at a steady state in vivo, and while more relevant than ex vivo studies, they are confounded by factors such as dietary or other physiological effects of the treatment, depending on patient-to-patient heterogeneity in response to the regimen.

If mTOR activation is confirmed as a potential mechanism of de novo resistance to enzalutamide, the addition of mTOR inhibitors to ER/AR blockade could be a feasible trial since combinations of enzalutamide and AR inhibitors with mTOR or PI3K inhibitors have proven clinical safety^40,41^. We recently completed accrual to a companion phase II trial (NCT02955394) of fulvestrant with or without enzalutamide in the neoadjuvant setting in women with T2 or greater ER+/HER2− BC, also with serial biopsies. The neoadjuvant trial will not have the confounding factor of the various prior treatments inherent to this metastatic trial. In conclusion, the present phase II study demonstrates that enzalutamide can be given safely in combination with fulvestrant for ER+ metastatic disease and that this combination warrants additional investigation. Future trials could be done in second-line endocrine therapy (progressing after AI and cdk4/6 inhibition) and in PI3K wild-type tumors to evaluate fulvestrant plus everolimus with or without an AR signaling blocker.

## Methods

### Study design and treatments

NCT02953860 (COMIRB 16–1001) was an open-label, non-randomized trial combining fulvestrant plus enzalutamide. All patients received fulvestrant 500 mg IM on days 1, 15, 29, and then every 4 weeks plus enzalutamide 160 mg po daily until disease progression or unacceptable toxicity. Pre- or peri-menopausal women received concurrent ovarian suppression with a gonadotropin-releasing hormone agonist.

### Study approval and ethics

The study was conducted at the University of Colorado and the University of Tennessee. The study protocol and its amendments were approved by the respective Institutional Review Boards, including the Colorado Multiple Institutional Review Board (COMIRB), the Office of Human Research Oversight (OHRO), and the University of Tennessee Institutional Review Board (UTHSC IRB). All patients provided written informed consent prior to participating in the study. The study was conducted under the principles of the World Medical Association, the Declaration of Helsinki, and the Good Clinical Practice guidelines of the International Conference on Harmonization. The study did not require an Investigational New Drug Application. Drug support (enzalutamide) was provided by Astellas and Pfizer as part of this investigator-sponsored research study.

### Study population

Eligible patients were women ≥18 years of age with adequate organ and bone marrow function and an ECOG performance score (PS) of 2 or less. All had metastatic BC determined to be ER-positive and HER2-negative. Prior anti-androgen treatment was not allowed. Prior fulvestrant was allowed if the treating physician felt that retreatment with fulvestrant was clinically indicated. The measurable or evaluable disease was required. Men were excluded due to potential confounding from androgenic stimuli. Central nervous system (CNS) metastases or a history of seizures were exclusionary due to the toxicity profile of enzalutamide. Determination of AR expression was not a requirement as it was expected that ~90% of tumors would stain for AR, and the assay has not yet been validated for clinical decision-making. Concomitant medications with substantial pharmacokinetic (PK) interaction with enzalutamide were avoided.

### Safety and antitumor assessment

All patients who received at least one dose of enzalutamide were assessed for safety biweekly for the first 4 weeks, then every 4 weeks until 30 days after the last dose of enzalutamide or prior to the initiation of a new treatment, whichever occurred first. Safety and tolerability were determined by assessment of AEs, physical examinations, ECOG PS, vital signs, and laboratory tests. The severity of abnormal laboratory values and AEs were classified using the National Cancer Institute Common Terminology Criteria for AEs (CTCAE), version 4.03. SAEs were also evaluated by Astellas Pharma Global Development—United States. A monthly teleconference was held among the institutional investigators to review patients and AEs. An institutional Data Safety and Monitoring Committee at the University of Colorado also had oversight for monitoring.

Radiographic assessments of disease status were performed at baseline and every 8 weeks thereafter. Tumor responses were defined using Response Evaluation Criteria in Solid Tumors (RECIST 1.1) version 1.1 criteria. Patients evaluable for response, PFS, and CBR had a follow-up scan or withdrew from the study because of toxicity or clinical progression.

### Tissue acquisition

Fresh tumor biopsies (punch biopsies for skin lesions or core needle biopsies for other sites) were required at study entry (baseline), and after 4 weeks on therapy (when both fulvestrant and enzalutamide were likely at steady state concentrations) and these were termed “WK5” biopsy. Archival tissue from the primary or prior biopsies of metastatic disease was obtained if available. A tumor biopsy at the time of progression was requested but is optional. Lithium-heparin (LiHep) plasma samples were obtained at the same intervals. After collection LiHep vacutainers were centrifuged for 20 min (600×*g*), separated plasma was transferred to a 15 mL conical tube, centrifuged a second time for 15 min (1500×*g*), aliquoted into 500 µL aliquots, and stored at −80 °C until analysis.

### Statistical analysis: sample size and data analysis considerations

CBR24 was used as the primary endpoint for sample size. Assuming the undesirable rate of 10% and the desired rate of 30%, a sample size of 24 provides 89% power to detect this 25% rate difference using an exact binomial test with a one-sided alpha of 0.085. Due to the exploratory nature of biomarker analyses, the type I error rate for all analyses was not adjusted for exploring multiple biomarkers. Of the 38 participants who consented to the clinical trial, 32 (84%) were eligible and evaluable, and 28 (74%) were considered evaluable for the CBR endpoint because they had at least one post-baseline tumor assessment. The 4 patients who withdrew early did not withdraw due to disease progression or for reasons of toxicity. Three (9.3%) participants had a fresh biopsy that stained negative for both AR and ER; they were thus excluded from the AR:ER ratio analysis. All missing observations were eliminated from the respective univariate analyses (Table 1).

### Clinical benefit rate and overall PFS

The clinical benefit rate was defined as the percent of patients with a response or stable disease by RECIST 1.1 criteria at the week 24 assessment (CBR24). A Kaplan–Meier survival curve was used to determine the median time to progression.

### Demographic and clinical risk factors of progression: univariate survival analysis

To identify the demographic and clinical risk factors of progression, the univariate relationships between PFS and each of the following risk factors were assessed with Cox Proportional Hazard models (CoxPH): age, ECOG PS, metastatic sites, bone metastatic sites ( ≥ 1 vs. 0; all vs. some/no sites), baseline and change in IHC Ki67, *ESR1*, and *PIK3CA/PTEN* mutation status, AR, ER, PR (score; ≥10% vs. <10% positive, continuous % positive), and the AR:ER ratio (<2 vs. ≥2). Additionally, the following lines of treatment were assessed: adjuvant chemotherapy, neoadjuvant chemotherapy, adjuvant endocrine therapy, chemotherapy for metastatic disease, endocrine therapy for metastatic disease, prior fulvestrant, the number of prior agents, the number of hormonal prior agents, and the number of non-hormonal prior agents.

### Multivariate Cox proportional hazard survival analysis

To assess the multivariate associations between risk factors and PFS, prior fulvestrant and all factors that had moderately significant (*p* < 0.2) univariate associations with PFS were included in a multivariate CoxPH model. Analyses were performed using R version 4.0.2.

### Mutation analyses methods

Core needle biopsies were acquired from patients who gave informed written consent, with ER^+^/HER2^−^ measurable or evaluable MBC without CNS disease. Formalin-fixed paraffin-embedded (FFPE) sections were analyzed for mutations in *ESR1* exon 8 and 67 other gene hotspots in genes frequently altered in cancer using a modified Archer VariantPlex Solid Tumor Assay through the CMOCO Laboratory (Department of Pathology, University of Colorado, Aurora, CO). The majority (60%) of biopsies were from the liver. The other sites included lymph nodes (16%), other soft tissue (9%), bone (9%), skin (3%), and breast (3%). Patients’ original primary tumors consisted of invasive ductal carcinomas (63%), invasive lobular carcinoma (18.5%) and invasive mammary carcinoma (14.8%), and unknown (3.7%).

### Immunohistochemistry

IHC was performed for ER (0.36 µg/mL clone 1D5 #M7047, Agilent/Dako, Santa Clara, CA), PR (0.5 µg/mL clone PgR 1294 #M3568, Agilent/Dako, Santa Clara, CA), AR (2.15 µg/mL clone 441 #M3562, Agilent/Dako, Santa Clara, CA), and GR (1:200 #3660 Cell Signaling Technologies, Danvers, MA) as well as Ki67 (0.2 µg/mL clone MIB-1 #M7240, Agilent/Dako, Santa Clara, CA) and cleaved caspase 3 (1:400 #9661, Cell Signaling Technologies, Danvers, MA) as described previously^7^. Briefly, biopsies from patients were formalin-fixed, paraffin-embedded, and 5 µm sections were heat immobilized onto charged slides. Slides were deparaffinized and rehydrated in a series of xylenes, and ethanols and antigens were heat retrieved using either citrate pH 6.0 (PR, AR, GR, Ki67) or 10 mM Tris/1 mM EDTA pH 9.0 buffer (ER, cleaved caspase 3) before probing with primary antibodies. Antibodies were detected using either the Vectastain Elite ABC Universal kit #PK-7200 (ER, PR, AR, Ki67) or the ImmPRESS Goat Anti-Rabbit IgG Polymer kit #MP-7451 (GR, cleaved caspase 3) from Vector Laboratories (Burlingame, CA) followed by DAB detection and counterstained with dilute hematoxylin.

### Reverse-phase protein microarray (RPPA)

Enriched epithelial cell subpopulations were isolated from 8 µm cryosections (>95% purity) using an Arcturus Pixcell IIe Laser Capture Microdissection system (Arcturus, Mountain View, CA, USA) as described^42^. Approximately 10,000 epithelial cells were captured for each sample. Microdissected material was lysed in extraction buffer composed of 1:1 Tissue Protein Extraction Reagent (TPER; ThermoFisher, Waltham, MA, USA) and 2× SDS-PAGE Sample Buffer (ThermoFisher) plus 2.5% beta-mercaptoethanol (BME) at a concentration of approximately 500–600 cells per 1 µL Samples were heated at 100 °C for 5 min, briefly centrifuged and stored at −20 °C until printed. Lysates were printed in triplicate spots (approx. 10 nL per spot) onto nitrocellulose coated slides (Grace Biolabs, Bend, OR, USA) using a Quanterix 2470 Arrayer (Quanterix, Billerica, MA, USA). Standard curves of control cell lysates were also included for quality assurance purposes^43^. Antibodies used on the arrays were validated before use^44^. Immunostaining was performed as previously described^45^. Each slide was probed with one primary antibody targeting each of the 158 proteins of interest (Supplementary Table 7). Biotinylated goat anti-rabbit (1:7500, Vector Laboratories Inc, Burlingame, CA) and rabbit anti-mouse (1:10, DakoCytomation, Carpinteria, CA, USA) IgG were used as secondary antibodies. Signal amplification was performed using a tyramide-based avidin/biotin amplification system (DakoCytomation, Carpinteria, CA, USA) followed by streptavidin-conjugated IRDye 680 (LI-COR, Lincoln, NE, USA) for visualization. Total protein was measured using Sypro Ruby protein blot staining per manufacturer’s instructions (Molecular Probes, Eugene, OR, USA). Images were acquired using a Tecan PowerScanner (Tecan, Mannedorf, Switzerland) and analyzed with MicroVigene Software Version 5.6. (Vigenetech, Carlisle, MA, USA). The final results represent negative control-subtracted and total protein normalized relative intensity values for each endpoint within a given patient sample.

To assess differences in phospho-protein abundance in pre-treatment tumor biopsies from patients who experienced “Short PFS” (defined in all RPPA analyses as PFS ≤ 60 days) compared to those who experienced “Long PFS” (PFS > 24 weeks), robust moderated t-tests by response were performed on the log_2_-transformed baseline abundance of each protein. An empirical Bayes method was employed to shrink sample variances toward a pooled estimate, allowing for a powerful and stable inference to detect significant differences in baseline expression between the Long PFS and Short PFS groups. The analysis was then repeated to compare differences in the change in abundance between baseline and the beginning of week 5 of treatment across PFS groups. Proteins in the mTOR pathway with significant baseline differences between “Long PFS” and “Short PFS” were identified and analyzed with previously described moderated t-tests to compare patients with *PTEN* and/or *PIK3CA* mutations (*PTEN*/*PIK3CA* mutants) to *PTEN*/*PIK3CA* wildtypes at baseline. Baseline and fold change associations between each RPPA phospho-protein, AR, and ER were then assessed. Each log_2_ autoscaled protein and AR were separately assessed using cell means linear regression models with baseline AR or ER as the outcome and the baseline protein of interest as the primary predictor. Each model was adjusted for post-treatment measurements. The association between the fold change of each protein and the fold change of AR or ER was similarly assessed, with the change in AR or ER as the outcome and the change in the protein of interest as the predictor.

We then evaluated whether proteins associated with AR and ER at baseline and with the change with treatment were enriched relative to all analyzed proteins. Proteins were first annotated with the biomaRt R package^46^, and proteins without annotations (18%) were eliminated. Candidate proteins were identified separately for each analysis (AR baseline, ER baseline, AR fold change, ER fold change) based on significance (*p* < 0.05). Gene Ontology (GO) enrichment analysis was then performed using the topGO R package^47^. Fisher’s exact tests with >2 proteins per node were used to test for enrichment.

#### Metabolomics analyses

Plasma metabolomics analyses were performed via UHPLC-MS (Vanquish-QExactive, Thermo Fisher), as previously described^48^. Briefly, plasma (20 µl) was extracted in 980 μL of methanol:acetonitrile:water (5:3:2, *v/v/v*). After vortexing at 4 °C for 30 min, extracts were separated from the protein pellet by centrifugation for 10 min at 18,000 g at 4 °C and stored at −80 °C until analysis. Analyses were performed using a Vanquish UHPLC coupled online to a Q Exactive mass spectrometer (Thermo Fisher, Bremen, Germany). Samples were analyzed using a 5 min gradient as described^48–50^. Solvents were supplemented with 0.1% formic acid for positive mode runs and 1 mM ammonium acetate for negative mode runs. MS acquisition, data analysis, and elaboration were performed as described^48–50^.

Baseline and week 5 Metabolomics data analysis was performed via MetaboAnalyst 5.0^51^ by comparing autoscale normalized data for “Short PFS” and “Long PFS” groups at baseline and after treatment. In addition, Log_2_-transformed autoscaled metabolomic data were analyzed analogously to RPPA data to compare patients who experienced “Short PFS” (defined for all metabolomic analyses as PFS < 24 weeks) with those who experienced “Long PFS” (PFS > 24 weeks) with the change following treatment.

### Reporting summary

Further information on research design is available in the Nature Research Reporting Summary linked to this article.

## Supplementary information

Supplementary Materials

Reporting Summary

## Data Availability

The laboratory data free of protected health information generated in this study are available upon request to the corresponding author.

## Code availability

Data cleaning and analyses were performed in R version 4.0.2 and Bioconductor. Code generated to analyze laboratory datasets free of protected health information is available upon reasonable request to the corresponding author.

## References

[1] van Hellemond IEG, Geurts SME, Tjan-Heijnen VCG (2018). Current status of extended adjuvant endocrine therapy in early stage breast cancer. Curr. Treat. Options Oncol..

[2] Collins LC (2011). Androgen receptor expression in breast cancer in relation to molecular phenotype: results from the Nurses’ Health Study. Mod. Pathol..

[3] D’Amato NC (2016). Cooperative dynamics of AR and ER activity in breast cancer. Mol. Cancer Res..

[4] Bleach R, McIlroy M (2018). The divergent function of androgen receptor in breast cancer; analysis of steroid mediators and tumor intracrinology. Front. Endocrinol..

[5] Hickey TE (2021). The androgen receptor is a tumor suppressor in estrogen receptor-positive breast cancer. Nat. Med..

[6] Rosas, E. et al. A positive feedback loop between TGFbeta and androgen receptor supports triple-negative breast cancer anoikis resistance. *Endocrinology* **162** (2021). 10.1210/endocr/bqaa226 PMC7806239 33294922

[7] Williams MM (2021). Steroid hormone receptor and infiltrating immune cell status reveals therapeutic vulnerabilities of ESR1-mutant breast cancer. Cancer Res..

[8] Barton VN (2017). Androgen receptor supports an anchorage-independent, cancer stem cell-like population in triple-negative breast cancer. Cancer Res..

[9] Cochrane DR (2014). Role of the androgen receptor in breast cancer and preclinical analysis of enzalutamide. Breast Cancer Res..

[10] De Amicis F (2010). Androgen receptor overexpression induces tamoxifen resistance in human breast cancer cells. Breast Cancer Res Treat..

[11] Cao L (2019). A high AR:ERalpha or PDEF:ERalpha ratio predicts a sub-optimal response to tamoxifen therapy in ERalpha-positive breast cancer. Cancer Chemother. Pharm..

[12] Rangel N (2018). The role of the AR/ER ratio in ER-positive breast cancer patients. Endocr. Relat. Cancer.

[13] Rangel, N. et al. AR/ER ratio correlates with expression of proliferation markers and with distinct subset of breast tumors. *Cells* **9** (2020). 10.3390/cells9041064 PMC7226480 32344660

[14] Gallicchio L, Macdonald R, Wood B, Rushovich E, Helzlsouer KJ (2011). Androgens and musculoskeletal symptoms among breast cancer patients on aromatase inhibitor therapy. Breast Cancer Res. Treat..

[15] Santen RJ (1982). In vivo and in vitro pharmacological studies of aminoglutethimide as an aromatase inhibitor. Cancer Res..

[16] Morris KT, Toth-Fejel S, Schmidt J, Fletcher WS, Pommier RF (2001). High dehydroepiandrosterone-sulfate predicts breast cancer progression during new aromatase inhibitor therapy and stimulates breast cancer cell growth in tissue culture: a renewed role for adrenalectomy. Surgery.

[17] Elliott KM (2014). Effects of aromatase inhibitors and body mass index on steroid hormone levels in women with early and advanced breast cancer. Br. J. Surg..

[18] Barton VN (2015). Multiple molecular subtypes of triple-negative breast cancer critically rely on androgen receptor and respond to enzalutamide in vivo. Mol. Cancer Ther..

[19] Gordon MA (2017). Synergy between androgen receptor antagonism and inhibition of mTOR and HER2 in breast cancer. Mol. Cancer Ther..

[20] Krop I (2020). A randomized placebo controlled phase II trial evaluating exemestane with or without enzalutamide in patients with hormone receptor-positive breast cancer. Clin. Cancer Res..

[21] Bleach R (2021). Steroid ligands, the forgotten triggers of nuclear receptor action; implications for acquired resistance to endocrine therapy. Clin. Cancer Res..

[22] Traina TA (2018). Enzalutamide for the treatment of androgen receptor-expressing triple-negative breast cancer. J. Clin. Oncol..

[23] Scher HI (2010). Antitumour activity of MDV3100 in castration-resistant prostate cancer: a phase 1-2 study. Lancet.

[24] Schwartzberg LS (2017). A phase I/Ib study of enzalutamide alone and in combination with endocrine therapies in women with advanced breast cancer. Clin. Cancer Res..

[25] Elias, A. et al. Abstract P1-16-05: MDV3100-08: A phase 1 study evaluating the safety and pharmacokinetics of enzalutamide plus fulvestrant in women with advanced hormone receptor-positive breast cancer. *Cancer Res.* **76**, P1-16-05–P11-16-05 (2016).

[26] Deleyto-Seldas N, Efeyan A (2021). The mTOR-autophagy axis and the control of metabolism. Front. Cell Dev. Biol..

[27] de la Cruz Lopez KG, Toledo Guzman ME, Sanchez EO, Garcia Carranca A (2019). mTORC1 as a regulator of mitochondrial functions and a therapeutic target in cancer. Front. Oncol..

[28] Zeng JD, Wu WKK, Wang HY, Li XX (2019). Serine and one-carbon metabolism, a bridge that links mTOR signaling and DNA methylation in cancer. Pharm. Res..

[29] Matikas A, Foukakis T, Bergh J (2018). RE: receptor conversion in distant breast cancer metastases: a systematic review and meta-analysis. J. Natl Cancer Inst..

[30] Schrijver W (2018). Receptor conversion in distant breast cancer metastases: a systematic review and meta-analysis. J. Natl Cancer Inst..

[31] Chen R (2014). The general amino acid control pathway regulates mTOR and autophagy during serum/glutamine starvation. J. Cell Biol..

[32] Bjorndal B (2018). Associations between fatty acid oxidation, hepatic mitochondrial function, and plasma acylcarnitine levels in mice. Nutr. Metab..

[33] Lehmann BD (2020). TBCRC 032 IB/II multicenter study: molecular insights to AR antagonist and PI3K inhibitor efficacy in patients with AR(+) metastatic triple-negative breast cancer. Clin. Cancer Res..

[34] Wheler JJ (2014). Unique molecular signatures as a hallmark of patients with metastatic breast cancer: implications for current treatment paradigms. Oncotarget.

[35] Kim JY (2017). Clinical implications of genomic profiles in metastatic breast cancer with a focus on TP53 and PIK3CA, the most frequently mutated genes. Oncotarget.

[36] Li H (2021). Activity of preclinical and phase I clinical trial of a novel androgen receptor antagonist GT0918 in metastatic breast cancer. Breast Cancer Res. Treat..

[37] Miller CP (2011). Design, synthesis, and preclinical characterization of the selective androgen receptor modulator (SARM) RAD140. ACS Med. Chem. Lett..

[38] Palmieri C (2021). Efficacy of enobosarm, a selective androgen receptor (AR) targeting agent, correlates with the degree of AR positivity in advanced AR+/estrogen receptor (ER)+ breast cancer in an international phase 2 clinical study. J. Clin. Oncol..

[39] Wardley A (2021). The efficacy and safety of enzalutamide with trastuzumab in patients with HER2+ and androgen receptor-positive metastatic or locally advanced breast cancer. Breast Cancer Res. Treat..

[40] Lehmann BD (2014). PIK3CA mutations in androgen receptor-positive triple negative breast cancer confer sensitivity to the combination of PI3K and androgen receptor inhibitors. Breast Cancer Res..

[41] Sarker D (2021). A phase I, open-label, dose-finding study of GSK2636771, a PI3Kbeta inhibitor, administered with enzalutamide in patients with metastatic castration-resistant prostate cancer. Clin. Cancer Res..

[42] Espina V (2006). Laser-capture microdissection. Nat. Protoc..

[43] Sheehan KM (2005). Use of reverse phase protein microarrays and reference standard development for molecular network analysis of metastatic ovarian carcinoma. Mol. Cell Proteom..

[44] Signore M, Reeder KA (2012). Antibody validation by Western blotting. Methods Mol. Biol..

[45] Pin E, Federici G, Petricoin EF (2014). Preparation and use of reverse protein microarrays. Curr. Protoc. Protein Sci..

[46] Durinck S, Spellman PT, Birney E, Huber W (2009). Mapping identifiers for the integration of genomic datasets with the R/Bioconductor package biomaRt. Nat. Protoc..

[47] Alexa, A. & Rahnenfurer, J. topGO: Enrichment Analysis for Gene Ontology. R package version 2.46.0. (2021).

[48] Nemkov T, Hansen KC, D’Alessandro A (2017). A three-minute method for high-throughput quantitative metabolomics and quantitative tracing experiments of central carbon and nitrogen pathways. Rapid Commun. Mass Spectrom..

[49] Reisz JA, Zheng C, D’Alessandro A, Nemkov T (2019). Untargeted and semi-targeted lipid analysis of biological samples using mass spectrometry-based metabolomics. Methods Mol. Biol..

[50] Johnson LC (2019). The plasma metabolome as a predictor of biological aging in humans. Geroscience.

[51] Pang Z (2021). MetaboAnalyst 5.0: narrowing the gap between raw spectra and functional insights. Nucleic Acids Res..

