# Supplementary material for: Phase II trial of fulvestrant plus enzalutamide in ER+/HER2− advanced breast cancer
Source: NPJ Breast Cancer. 2023 May 20;9:41. doi: 10.1038/s41523-023-00544-z (PMC10199936; doi:10.1038/s41523-023-00544-z)
Supplement: Supplementary file 1 — Supplementary Materials [file 41523_2023_544_MOESM1_ESM.pdf]

Supplementary Table 1: The phospho-proteins from pretreatment tissue (at baseline) that had significantly ($p<0.05$) different expression across the Long versus Short PFS groups according to the log2 moderated t-test

Protein	Long PFS Mean (SD)[b]	Short PFS Mean (SD)[b]	Mean Diff[a]	t-test p-value[c]
mTOR.S2448	14.27 (0.91)	15.29 (0.64)	-1.02	**0.008**
HLA.DR.DP.DQ.DX.total	11.54 (0.14)	12.76 (1.29)	-1.22	**0.021**
eNOS.NOSIII.S116	14.17 (0.33)	14.71 (0.52)	-0.54	**0.029**
S6RP.S240.S244	10.57 (1.45)	12.53 (2.04)	-1.96	**0.031**
eIF4G.S1108	13.55 (1.32)	14.74 (1.07)	-1.19	**0.037**
H2A.X.S139	14.24 (0.69)	14.9 (0.6)	-0.66	**0.04**
p70S6K.T389	13.71 (0.98)	14.66 (0.95)	-0.95	**0.043**
MET.Y1234.Y1235	11.51 (1.24)	12.86 (1.51)	-1.35	0.05
PRAS40.T246	10.99 (5.03)	13.29 (4.11)	-2.30	0.264

[a]Difference is defined as the mean baseline expression amongst patients in the “Long PFS” group minus the mean baseline expression amongst patients in the “Short PFS” group. A positive difference indicates that the Long PFS group had a larger mean baseline expression than the Short PFS group. A negative difference indicates that the Short PFS group had a larger mean baseline expression than the Long PFS group. The corresponding p-values indicate whether this difference in baseline expression was statistically significant. [b]Means and SDs are reported in $\log_2$ units. Medians, minimums, and maximums are reported in original units. [c]P-value from $\log_2$ moderated t-test.

Supplementary Table 2: Differentially (p<0.2) detected proteins between baseline and week 5 of treatment in the Short PFS versus Long PFS groups according to the log2 moderated t-test.

Protein	Long PFS Mean Change (SD)[1]	Short PFS Mean Change (SD)[1]	LogFC	t-test p-value[2]
cABL.Y245	1.57 (3.9)	-3.1 (4.69)	4.68	**0.03**
LKB1.S334	0.35 (0.3)	-0.03 (0.28)	0.38	**0.037**
PAK1.S199.S204.PAK2.S192.S197	0.42 (0.59)	-0.56 (1.08)	0.98	**0.037**
S6RP.S240.S244	1.48 (1.69)	-0.21 (1.72)	1.69	**0.042**
eIF4G.S1108	0.76 (1.26)	-0.29 (1.3)	1.05	0.091
EGFR.total	0.35 (0.53)	-0.12 (0.69)	0.47	0.141
RB.S780	-1.25 (5.06)	2.31 (5.21)	-3.56	0.141
S6RP.S235.S236	1.21 (1.83)	-0.04 (1.86)	1.25	0.152
cKIT.Y719	0.27 (0.29)	-0.08 (0.59)	0.35	0.182
TROP2.total	-0.72 (2.1)	0.23 (1.16)	-0.95	0.19
ATP.Citrate.Lyase.S454	0.34 (0.56)	-0.02 (0.53)	0.36	0.193
mTOR.S2448	0.41 (1.14)	-0.14 (0.71)	0.55	0.195
STAT1.Y701	0.37 (0.68)	-0.39 (1.42)	0.75	0.196

[1]Change is defined as the difference between baseline and week 5 of treatment; a positive change indicates a greater expression at week 5 than at baseline and a negative change indicates a greater expression at baseline than at week 5. All values are in $\log_2$ units.[2]P-value from $\log_2$ moderated t-test.

Supplementary Table 3a. Baseline associations between proteins and ER

Significant (p<0.05) baseline associations between each protein of interest and ER are presented. Cell means linear regression models were run separately for each protein to assess its baseline correlation with ER. Each model adjusted for week 5 measurements of ER and the protein of interest. The estimates and 95% confidence intervals are in terms of normalized autoscaled units. Positive estimates indicate a positive correlation with ER and negative estimates indicate a negative correlation with ER. Greater absolute values of estimates indicate stronger correlations. For example, BCL2.S70 is significantly correlated with ER at baseline; each normalized autoscaled unit increase in baseline BCL2.S70 is associated with a 0.59 (95% CI: 0.36 to 0.81) unit increase in baseline ER, after adjusting for week5 AR and week5 BCL2.S70 (p<0.001).

Protein	Est.(CI)	p
Progesterone.Rec.S190	3.45 (1.07, 5.84)	0.01
Cyclin.D1.total	1.12 (0.71, 1.54)	<0.001
PRK1.T774.PRK2.T816	0.72 (0.42, 1.03)	<0.001
FOXO1.T24.FOXO3a.T32	0.69 (0.3, 1.09)	0.003
PKA.C.T197	0.69 (0.38, 1.01)	<0.001
Caspase.3..cleaved.D175	0.68 (0.45, 0.91)	<0.001
HER3.total	0.67 (0.41, 0.93)	<0.001
p53.total	0.66 (0.37, 0.96)	<0.001
HER4.total	0.66 (0.4, 0.92)	<0.001
Estrogen.Rec.alpha.S118	0.66 (0.47, 0.85)	<0.001
Histone.H3.S10	0.64 (0.33, 0.94)	<0.001
CREB.S133	0.63 (0.34, 0.93)	<0.001
PP2A.alpha.subunit.total	0.63 (0.4, 0.85)	<0.001
SRC.Y527	0.63 (0.07, 1.18)	0.04
cPLA2.S505	0.62 (0.34, 0.91)	<0.001
IRS1.S612	0.62 (0.29, 0.96)	0.002
PDK1.S241	0.62 (0.24, 0.99)	0.004
Androgen.Rec.S81	0.61 (0.34, 0.88)	<0.001
NFkB.p65.S536	0.6 (0.33, 0.86)	<0.001
Cyclin.B1.total	0.6 (0.26, 0.94)	0.003
B.RAF.S445	0.59 (0.27, 0.92)	0.002
MEK1.2.S217.S221	0.59 (0.33, 0.85)	<0.001
BCL2.S70	0.59 (0.36, 0.81)	<0.001
CHK1.S345	0.58 (0.29, 0.88)	<0.001
PLCgamma1.Y783	0.58 (0.28, 0.89)	0.001
RAF.S259	0.58 (0.27, 0.9)	0.002
MLH1.total	0.58 (0.27, 0.88)	0.002
PAK1.T423.PAK2.T402	0.58 (0.29, 0.86)	<0.001
FOXO1.S256	0.57 (0.27, 0.88)	0.001
PDGFRb.Y751	0.57 (0.12, 1.02)	0.02
IkBa.S32.S36	0.56 (0.25, 0.87)	0.002
FADD.S194	0.55 (0.31, 0.8)	<0.001
STAT3.S727	0.55 (0.26, 0.85)	0.002
SGK1.S78	0.55 (0.26, 0.84)	0.002
Aurora.A.T288.B.T232.C.T198	0.55 (0.18, 0.92)	0.01

Androgen.Rec.V7.total	0.55 (0.2, 0.89)	0.006
SMAD2.S245.S250.S255	0.54 (0.26, 0.82)	0.001
eIF4E.S209	0.54 (0.24, 0.83)	0.002
MSK1.S360	0.53 (0.22, 0.84)	0.003
eNOS.S1177	0.53 (0.24, 0.82)	0.002
C.RAF.S338	0.53 (0.22, 0.84)	0.003
HER2.total	0.53 (0.25, 0.81)	0.001
MSH2.total	0.52 (0.19, 0.86)	0.006
Catenin..beta.S33.S37.T41	0.52 (0.23, 0.82)	0.003
MDM2.S166	0.52 (0.23, 0.81)	0.002
YAP.S127	0.51 (0.21, 0.8)	0.003
RSK3.T356.S360	0.51 (0.06, 0.96)	0.04
TROP2.total	0.5 (0.13, 0.86)	0.02
EGFR.Y1173	0.5 (0.17, 0.82)	0.007
M.CSF.Rec.Y723	0.49 (0.23, 0.75)	0.002
HLA.DR.total	0.49 (0.14, 0.83)	0.01
ERK1.2.T202.Y204	0.48 (0.11, 0.86)	0.02
p90RSK.S380	0.48 (0.12, 0.84)	0.02
PARP..cleaved.D214	0.48 (0.04, 0.92)	0.04
p70S6K.S371	0.47 (0.16, 0.79)	0.009
FOXM1.T600	0.47 (0.15, 0.8)	0.01
RAS.GRF1.S916	0.47 (0.16, 0.78)	0.008
Androgen.Rec.total	0.46 (0.08, 0.85)	0.03
eNOS.NOSIII.S116	0.46 (0.13, 0.78)	0.01
CD3.epsilon.total	0.46 (0.15, 0.76)	0.009
STAT3.Y705	0.45 (0.12, 0.78)	0.02
GSK3aB.S21.S9	0.45 (0.11, 0.79)	0.02
X4EBP1.S65	0.45 (0.11, 0.79)	0.02
ELK1.S383	0.44 (0.11, 0.78)	0.02
BAD.S136	0.44 (0.1, 0.77)	0.02
ATR.S428	0.42 (0.09, 0.75)	0.02
Caspase.9..cleaved.D330	0.42 (0.16, 0.68)	0.006
Insulin.Rec.beta.total	0.41 (0.07, 0.76)	0.03
Catenin..beta.T41.S45	0.38 (0.06, 0.7)	0.03
RB.S780	0.37 (0.06, 0.69)	0.03
eNOS.S113	0.37 (0.04, 0.7)	0.04
HER3.Y1289	0.37 (0.03, 0.71)	0.05
ATM.S1981	-0.42 (-0.81, -0.03)	0.05
MSH6.total	-1.04 (-1.83, -0.25)	0.02

Supplementary Table 3b. Fold change associations between proteins and ER

Proteins with fold changes that were significantly (p<0.05) associated with the change in ER from baseline to week 5 of treatment are presented. Cell means linear regression models were run separately for each protein to assess the association between the fold change in each protein with the fold change in ER. The estimates and 95% confidence intervals are in terms of normalized autoscaled units. Positive estimates indicate a positive correlation with ER and negative estimates indicate a negative correlation with ER. Greater absolute values of estimates indicate stronger correlations. For example, the change in AMPK.alpha.S485 from baseline to week 5 is significantly correlated with the change in ER from baseline to week 5; each normalized autoscaled unit increase in the fold change of AMPK.alpha.S485 is associated with a 0.35 (95% CI: 0.11 to 0.59) unit increase in the fold change of ER (p=0.009).

Protein	Est.(CI)	p
Progesterone.Rec.S190	3.47 (0.79, 6.16)	0.02
Cyclin.D1.total	0.97 (0.44, 1.49)	0.002
X4EBP1.T70	0.72 (0.2, 1.23)	0.01
FOXO1.T24.FOXO3a.T32	0.71 (0.39, 1.03)	<0.001
HER3.total	0.71 (0.48, 0.93)	<0.001
HER4.total	0.7 (0.45, 0.95)	<0.001
p53.total	0.68 (0.42, 0.94)	<0.001
SRC.Y527	0.67 (0.16, 1.18)	0.02
PKA.C.T197	0.64 (0.4, 0.88)	<0.001
Estrogen.Rec.alpha.S118	0.64 (0.48, 0.8)	<0.001
eIF4E.S209	0.63 (0.34, 0.92)	<0.001
Caspase.3..cleaved.D175	0.63 (0.46, 0.79)	<0.001
Histone.H3.S10	0.62 (0.37, 0.87)	<0.001
IkBa.S32.S36	0.61 (0.35, 0.87)	<0.001
CREB.S133	0.6 (0.34, 0.85)	<0.001
IRS1.S612	0.58 (0.3, 0.86)	<0.001
PRK1.T774.PRK2.T816	0.58 (0.25, 0.9)	0.002
PDGFRb.Y751	0.56 (0.21, 0.91)	0.005
HER2.total	0.56 (0.3, 0.81)	<0.001
Androgen.Rec.S650	0.55 (0.17, 0.94)	0.01
PARP..cleaved.D214	0.55 (0.16, 0.95)	0.01
Catenin..beta.S33.S37.T41	0.55 (0.31, 0.79)	<0.001
BCL2.S70	0.55 (0.38, 0.72)	<0.001
Aurora.A.T288.B.T232.C.T198	0.54 (0.18, 0.89)	0.007
MDM2.S166	0.53 (0.31, 0.75)	<0.001
RAF.S259	0.53 (0.24, 0.81)	0.002
C.RAF.S338	0.53 (0.27, 0.78)	<0.001
Insulin.Rec.beta.total	0.52 (0.22, 0.83)	0.003
MLH1.total	0.52 (0.24, 0.8)	0.002
PP2A.alpha.subunit.total	0.52 (0.29, 0.74)	<0.001
Cyclin.B1.total	0.51 (0.26, 0.77)	<0.001
MSH2.total	0.51 (0.19, 0.83)	0.005
PLCgamma1.Y783	0.51 (0.25, 0.76)	<0.001

PDK1.S241	0.5 (0.11, 0.9)	0.02
HLA.DR.total	0.5 (0.26, 0.73)	<0.001
CHK1.S345	0.49 (0.23, 0.76)	0.001
eNOS.NOSIII.S116	0.49 (0.23, 0.75)	0.001
MSK1.S360	0.49 (0.24, 0.74)	0.001
M.CSF.Rec.Y723	0.48 (0.31, 0.65)	<0.001
YAP.S127	0.48 (0.25, 0.71)	<0.001
RAS.GRF1.S916	0.48 (0.25, 0.71)	<0.001
STAT3.Y705	0.47 (0.21, 0.74)	0.002
EGFR.Y1173	0.47 (0.21, 0.72)	0.002
BAD.S136	0.47 (0.2, 0.74)	0.003
Caspase.9..cleaved.D330	0.47 (0.3, 0.63)	<0.001
cPLA2.S505	0.46 (0.16, 0.76)	0.006
p70S6K.S371	0.46 (0.2, 0.73)	0.003
Androgen.Rec.S81	0.46 (0.21, 0.71)	0.002
FOXO1.S256	0.45 (0.19, 0.72)	0.003
Androgen.Rec.total	0.45 (0.09, 0.82)	0.02
SGK1.S78	0.45 (0.22, 0.68)	0.001
GSK3aB.S21.S9	0.45 (0.16, 0.74)	0.007
Catenin..beta.T41.S45	0.45 (0.2, 0.69)	0.002
STAT3.S727	0.44 (0.18, 0.71)	0.004
eNOS.S1177	0.44 (0.19, 0.68)	0.002
EGFR.total	0.43 (0.07, 0.8)	0.03
Androgen.Rec.V7.total	0.43 (0.2, 0.66)	0.002
p90RSK.S380	0.43 (0.11, 0.74)	0.02
HER4.Y1284	0.43 (0.19, 0.66)	0.002
BAD.S112	0.42 (0.14, 0.7)	0.008
p90RSK.T359.S360	0.41 (0.14, 0.68)	0.007
HSP90.T5.T7	0.41 (0.08, 0.74)	0.02
NFkB.p65.S536	0.4 (0.19, 0.62)	0.001
FADD.S194	0.4 (0.16, 0.65)	0.004
CD3.epsilon.total	0.4 (0.18, 0.63)	0.002
TYK2.T1054.Y1055	0.4 (0.09, 0.72)	0.02
PAK1.T423.PAK2.T402	0.4 (0.09, 0.71)	0.02
SMAD2.S245.S250.S255	0.39 (0.12, 0.67)	0.01
ELK1.S383	0.38 (0.09, 0.67)	0.02
NRF2.total	0.38 (0.17, 0.58)	0.002
X4EBP1.S65	0.38 (0.07, 0.68)	0.02
ATR.S428	0.37 (0.09, 0.66)	0.02
B.RAF.S445	0.37 (0.03, 0.71)	0.04
MEK1.2.S217.S221	0.36 (0.1, 0.63)	0.01
FOXM1.T600	0.36 (0.11, 0.61)	0.01
H2A.X.S139	0.36 (0.09, 0.62)	0.02
AMPK.alpha.S485	0.35 (0.11, 0.59)	0.009
Cyclin.A.total	0.35 (0.03, 0.67)	0.05

HER3.Y1289	0.34 (0.09, 0.6)	0.02
cKIT.Y719	0.32 (0.05, 0.59)	0.03
eNOS.S113	0.3 (0.05, 0.56)	0.03
STAT4.Y693	0.3 (0.03, 0.56)	0.04
Acetyl.CoA.Carboxylase.S79	0.28 (0.03, 0.52)	0.04
PDGFRb.Y716	0.27 (0.03, 0.5)	0.04
Tuberin.TSC2.Y1571	0.25 (0.03, 0.48)	0.04
MSH6.total	-1.28 (-2.03, -0.53)	0.003

Supplementary Table 4a. Baseline associations between proteins and AR

Significant (p<0.05) baseline associations between each protein of interest and AR are presented. Cell means linear regression models were run separately for each protein to assess its baseline correlation with AR. Each model adjusted for week 5 measurements of AR and the protein of interest. The estimates and 95% confidence intervals are in terms of normalized autoscaled units. Positive estimates indicate a positive correlation with AR and negative estimates indicate a negative correlation with AR. Greater absolute values of estimates indicate stronger correlations. For example, ARR.S428 is significantly correlated with AR at baseline; each normalized autoscaled unit increase in baseline ATR.S42 is associated with a 0.54 (95% CI: 0.25 to 0.83) unit increase in baseline AR, after adjusting for week 5 AR and week 5 ATR.S42 (p=0.002).

Protein	Est.(CI)	p
SRC.Y527	0.76 (0.26, 1.26)	0.008
Cyclin.D1.total	0.72 (0.07, 1.37)	0.04
HER4.total	0.66 (0.33, 0.98)	<0.001
Ki67.total	0.64 (0.04, 1.24)	0.05
HER3.total	0.61 (0.3, 0.92)	0.001
Androgen.Rec.S81	0.6 (0.36, 0.84)	<0.001
PRK1.T774.PRK2.T816	0.6 (0.27, 0.92)	0.002
PDK1.S241	0.6 (0.18, 1.01)	0.01
MLH1.total	0.58 (0.3, 0.86)	<0.001
cPLA2.S505	0.56 (0.25, 0.87)	0.002
MSH2.total	0.55 (0.19, 0.92)	0.007
MEK1.2.S217.S221	0.55 (0.3, 0.81)	<0.001
PKA.C.T197	0.55 (0.27, 0.83)	0.001
RAF.S259	0.54 (0.29, 0.8)	<0.001
ATR.S428	0.54 (0.25, 0.83)	0.002
p53.total	0.5 (0.2, 0.79)	0.004
MSK1.S360	0.5 (0.16, 0.83)	0.009
ASK1.S83	0.49 (0.15, 0.83)	0.01
Aurora.A.T288.B.T232.C.T198	0.49 (0.1, 0.89)	0.02
RB.S780	0.49 (0.2, 0.78)	0.004
NFkB.p65.S536	0.49 (0.26, 0.72)	<0.001
PARP..cleaved.D214	0.49 (0.06, 0.92)	0.04
Estrogen.Rec.alpha.total	0.49 (0.08, 0.89)	0.03
HER2.total	0.49 (0.18, 0.8)	0.006
X4EBP1.S65	0.48 (0.15, 0.81)	0.01
FADD.S194	0.46 (0.18, 0.74)	0.005
PP2A.alpha.subunit.total	0.46 (0.17, 0.75)	0.006
FOXO1.T24.FOXO3a.T32	0.46 (0.03, 0.88)	0.05
FOXO1.S256	0.43 (0.18, 0.68)	0.003
mTOR.S2448	0.43 (0.11, 0.75)	0.02
Catenin..beta.S33.S37.T41	0.42 (0.11, 0.74)	0.02
p70S6K.S371	0.42 (0.11, 0.73)	0.01

PRAS40.T246	0.42 (0.09, 0.75)	0.02
YAP.S127	0.42 (0.15, 0.69)	0.006
HSP90.T5.T7	0.42 (0.07, 0.76)	0.03
GAB1.Y627	0.4 (0.04, 0.76)	0.04
p70S6K.T412	0.4 (0.03, 0.77)	0.05
BCL2.S70	0.39 (0.05, 0.74)	0.04
GSK3aB.S21.S9	0.39 (0.05, 0.74)	0.04
STAT3.S727	0.37 (0.03, 0.72)	0.04
eNOS.S1177	0.37 (0.11, 0.64)	0.01
p90RSK.S380	0.37 (0.05, 0.69)	0.03
EGFR.Y1173	0.37 (0.04, 0.7)	0.04
eIF4E.S209	0.37 (0.06, 0.68)	0.03
H2A.X.S139	0.36 (0.1, 0.63)	0.01
SMAD2.S245.S250.S255	0.36 (0.04, 0.68)	0.04
eNOS.NOSIII.S116	0.35 (0.11, 0.6)	0.01
MDM2.S166	0.34 (0.05, 0.63)	0.03

Supplementary Table 4b. Fold change associations between proteins and AR

Proteins with fold changes that were significantly (p<0.05) associated with the change in AR from baseline to the start of week 5 of treatment are presented. Cell means linear regression models were run separately for each protein to assess the association between the fold change in each protein with the fold change in AR. The estimates and 95% confidence intervals are in terms of normalized autoscaled units. Positive estimates indicate a positive correlation with AR and negative estimates indicate a negative correlation with AR. Greater absolute values of estimates indicate stronger correlations. For example, the change in AMPK.alpha.S485 from baseline to week 5 is significantly correlated with the change in AR from baseline to week 5; each normalized autoscaled unit increase in the fold change of AMPK.alpha.S485 is associated with a 0.48 (95% CI: 0.27 to 0.69) unit increase in the fold change of AR (p<0.001).

Protein	Est.(CI)	p
SRC.Y527	0.93 (0.47, 1.38)	<0.001
Cyclin.D1.total	0.84 (0.25, 1.43)	0.01
Ki67.total	0.84 (0.2, 1.48)	0.02
HER4.total	0.69 (0.42, 0.97)	<0.001
HER3.total	0.68 (0.41, 0.94)	<0.001
RAF.S259	0.67 (0.43, 0.91)	<0.001
Aurora.A.T288.B.T232.C.T198	0.66 (0.33, 1)	<0.001
p53.total	0.65 (0.37, 0.94)	<0.001
X4EBP1.T70	0.64 (0.09, 1.2)	0.03
MSH2.total	0.64 (0.35, 0.93)	<0.001
PRK1.T774.PRK2.T816	0.64 (0.32, 0.96)	<0.001
MLH1.total	0.63 (0.37, 0.88)	<0.001
cPLA2.S505	0.62 (0.36, 0.88)	<0.001
Androgen.Rec.S81	0.62 (0.42, 0.81)	<0.001
PKA.C.T197	0.61 (0.34, 0.88)	<0.001
FOXO1.S256	0.6 (0.38, 0.82)	<0.001
S6RP.S240.S244	0.59 (0.18, 1)	0.01
p70S6K.T412	0.59 (0.24, 0.93)	0.003
EGFR.total	0.59 (0.25, 0.92)	0.003
S6RP.S235.S236	0.58 (0.14, 1.01)	0.02
PDK1.S241	0.57 (0.18, 0.97)	0.01
eNOS.NOSIII.S116	0.57 (0.33, 0.81)	<0.001
mTOR.S2448	0.56 (0.25, 0.87)	0.002
HER2.total	0.55 (0.28, 0.82)	<0.001
MEK1.2.S217.S221	0.55 (0.34, 0.76)	<0.001
YAP.S127	0.55 (0.33, 0.76)	<0.001
FADD.S194	0.54 (0.33, 0.74)	<0.001
PP2A.alpha.subunit.total	0.53 (0.3, 0.76)	<0.001
EGFR.Y1173	0.53 (0.29, 0.78)	<0.001
p90RSK.S380	0.53 (0.23, 0.83)	0.003
X4EBP1.S65	0.53 (0.26, 0.8)	0.001

GSK3aB.S21.S9	0.52 (0.25, 0.8)	0.001
eNOS.S1177	0.52 (0.3, 0.75)	<0.001
Catenin..beta.S33.S37.T41	0.52 (0.26, 0.78)	<0.001
Insulin.Rec.beta.total	0.52 (0.2, 0.84)	0.005
ATR.S428	0.52 (0.26, 0.77)	<0.001
NFkB.p65.S536	0.51 (0.33, 0.69)	<0.001
p70S6K.S371	0.51 (0.24, 0.77)	0.001
PAK1.T423.PAK2.T402	0.5 (0.21, 0.8)	0.003
eIF4E.S209	0.49 (0.19, 0.8)	0.005
Estrogen.Rec.alpha.total	0.48 (0.09, 0.87)	0.02
AMPK.alpha.S485	0.48 (0.27, 0.69)	<0.001
Cyclin.B1.total	0.48 (0.2, 0.76)	0.003
FOXO1.T24.FOXO3a.T32	0.47 (0.07, 0.88)	0.03
BAD.S112	0.47 (0.2, 0.75)	0.003
Estrogen.Rec.alpha.S118	0.47 (0.21, 0.73)	0.002
SMAD2.S245.S250.S255	0.46 (0.2, 0.73)	0.003
MDM2.S166	0.46 (0.2, 0.72)	0.002
FOXM1.T600	0.44 (0.2, 0.68)	0.002
BAD.S136	0.44 (0.15, 0.73)	0.007
Catenin..beta.T41.S45	0.44 (0.18, 0.7)	0.003
ELK1.S383	0.43 (0.15, 0.72)	0.007
C.RAF.S338	0.43 (0.13, 0.73)	0.01
STAT3.S727	0.43 (0.14, 0.72)	0.008
H2A.X.S139	0.43 (0.17, 0.69)	0.004
PLCgamma1.Y783	0.43 (0.14, 0.72)	0.009
eIF4G.S1108	0.42 (0.12, 0.71)	0.01
RAS.GRF1.S916	0.41 (0.15, 0.68)	0.006
IRS1.S612	0.4 (0.05, 0.75)	0.03
HER4.Y1284	0.4 (0.14, 0.66)	0.007
B.RAF.S445	0.39 (0.04, 0.74)	0.04
RB.S780	0.39 (0.14, 0.63)	0.005
HSP90.T5.T7	0.37 (0.05, 0.68)	0.03
cABL.T735	0.37 (0.11, 0.63)	0.01
HLA.DR.total	0.36 (0.08, 0.65)	0.02
SRC.Family.Y416	0.36 (0.06, 0.67)	0.03
SAPK.JNK.T183.Y185	0.36 (0.08, 0.65)	0.02
TYK2.T1054.Y1055	0.36 (0.02, 0.7)	0.05
AMPK.alpha.T172	0.35 (0.12, 0.58)	0.008
HER3.Y1289	0.34 (0.08, 0.61)	0.02
PP2A.beta.subunit.total	0.34 (0.05, 0.63)	0.03
ALK.Y1586	0.34 (0.07, 0.6)	0.02
STAT3.Y705	0.34 (0.02, 0.65)	0.05
NRF2.total	0.34 (0.11, 0.56)	0.009

eNOS.S113	0.33 (0.07, 0.59)	0.02
Acetyl.CoA.Carboxylase.S79	0.33 (0.08, 0.57)	0.02
BCL2.S70	0.33 (0.06, 0.59)	0.02
Androgen.Rec.V7.total	0.32 (0.05, 0.59)	0.03
Caspase.9..cleaved.D330	0.3 (0.06, 0.53)	0.02
MSH6.total	-1.02 (-1.87, -0.17)	0.03

Supplementary Table 5a. Enriched pathways among proteins significantly associated with ER at baseline

GO.ID	Term	Significant	Expected	p
GO:0010628	positive regulation of gene expression	22	21.5	0.0027
GO:0035556	intracellular signal transduction	47	45.84	0.0037
GO:0032355	response to estradiol	8	5.66	0.0043
GO:0048538	thymus development	7	4.53	0.0094
GO:0031647	regulation of protein stability	6	4.53	0.0118
GO:0050679	positive regulation of epithelial cell proliferation	7	6.79	0.0125
GO:0008285	negative regulation of cell population proliferation	14	12.45	0.0142
GO:0000122	negative regulation of transcription by RNA polymerase II	11	9.05	0.0202
GO:0008584	male gonad development	6	3.96	0.0212
GO:1902533	positive regulation of intracellular signal transduction	19	20.37	0.0237
GO:0016485	protein processing	7	3.96	0.025
GO:0006915	apoptotic process	36	36.22	0.0265
GO:2000779	regulation of double-strand break repair	4	2.26	0.0287
GO:0010165	response to X-ray	4	2.26	0.0287
GO:0030878	thyroid gland development	4	2.26	0.0287
GO:1903798	regulation of production of miRNAs involved in gene silencing by miRNA	4	2.26	0.0287
GO:0060324	face development	4	2.26	0.0287
GO:0045840	positive regulation of mitotic nuclear division	4	2.26	0.0287
GO:0032872	regulation of stress-activated MAPK cascade	5	3.96	0.0293
GO:0051384	response to glucocorticoid	6	5.66	0.0298
GO:0045596	negative regulation of cell differentiation	11	8.49	0.0371
GO:0048646	anatomical structure formation involved in morphogenesis	13	16.98	0.0395
GO:0001701	in utero embryonic development	9	7.36	0.0417
GO:2000113	negative regulation of cellular macromolecule biosynthetic process	20	15.84	0.0427
GO:0006468	protein phosphorylation	31	34.52	0.0455
GO:0071480	cellular response to gamma radiation	5	3.4	0.0462

Pathways significantly ($p<0.05$) enriched in proteins significantly associated with RPPA ER at baseline are presented. Enrichment was assessed with Fisher’s exact test.

Supplementary Table 5b. Enriched pathways among proteins significantly associated with RRPA ER, evaluated at the change with treatment

GO.ID	Term	Significant	Expected	p
GO:0008584	male gonad development	7	4.29	0.003
GO:0032355	response to estradiol	9	6.12	0.0062
GO:0035556	intracellular signal transduction	48	49.6	0.0126
GO:0035019	somatic stem cell population maintenance	5	3.06	0.0166
GO:0045747	positive regulation of Notch signaling pathway	5	3.06	0.0166
GO:0031647	regulation of protein stability	6	4.9	0.0171
GO:0065003	protein-containing complex assembly	13	14.7	0.0173
GO:0010628	positive regulation of gene expression	23	23.27	0.0178
GO:0050679	positive regulation of epithelial cell proliferation	7	7.35	0.0184
GO:0008150	biological_process	58	61.85	0.0345
GO:0008284	positive regulation of cell population proliferation	26	27.56	0.0354
GO:0000122	negative regulation of transcription by RNA polymerase II	11	9.8	0.0379
GO:0045844	positive regulation of striated muscle tissue development	4	2.45	0.0385
GO:2000779	regulation of double-strand break repair	4	2.45	0.0385
GO:1903798	regulation of production of miRNAs involved in gene silencing by miRNA	4	2.45	0.0385
GO:0046627	negative regulation of insulin receptor signaling pathway	4	2.45	0.0385
GO:0051592	response to calcium ion	4	2.45	0.0385
GO:0051384	response to glucocorticoid	6	6.12	0.0405
GO:0071248	cellular response to metal ion	5	4.9	0.0408

Pathways significantly (p<0.05) enriched in proteins significantly associated with RPPA AR when evaluated at the change in treatment baseline to week5 are presented. Enrichment was assessed with Fisher’s exact test.

Supplementary Table 6a. Enriched pathways among proteins significantly associated with RRPA AR at baseline

GO.ID	Term	Annotated	Significant	Expected	p
GO:0000122	negative regulation of transcription by RNA polymerase II	16	10	6.33	0.0021
GO:0045596	negative regulation of cell differentiation	15	10	5.93	0.0043
GO:1903798	regulation of production of miRNAs involved in gene silencing by miRNA	4	4	1.58	0.0054
GO:0003179	heart valve morphogenesis	4	4	1.58	0.0054
GO:2001237	negative regulation of extrinsic apoptotic signaling pathway	9	5	3.56	0.0198
GO:0010628	positive regulation of gene expression	38	16	15.02	0.0199
GO:0010611	regulation of cardiac muscle hypertrophy	3	3	1.19	0.0204
GO:0001942	hair follicle development	3	3	1.19	0.0204
GO:0032757	positive regulation of interleukin-8 production	3	3	1.19	0.0204
GO:0034660	ncRNA metabolic process	3	3	1.19	0.0204
GO:0072132	mesenchyme morphogenesis	3	3	1.19	0.0204
GO:0017148	negative regulation of translation	3	3	1.19	0.0204
GO:1900371	regulation of purine nucleotide biosynthetic process	3	3	1.19	0.0204
GO:0003197	endocardial cushion development	3	3	1.19	0.0204
GO:0090263	positive regulation of canonical Wnt signaling pathway	3	3	1.19	0.0204
GO:2000637	positive regulation of gene silencing by miRNA	3	3	1.19	0.0204
GO:0071168	protein localization to chromatin	3	3	1.19	0.0204
GO:0001568	blood vessel development	22	8	8.7	0.0208
GO:0045747	positive regulation of Notch signaling pathway	5	4	1.98	0.0213
GO:0050679	positive regulation of epithelial cell proliferation	12	5	4.74	0.0235
GO:0035556	intracellular signal transduction	81	29	32.02	0.0238
GO:0051240	positive regulation of multicellular organismal process	46	19	18.19	0.0259
GO:0045893	positive regulation of transcription, DNA-templated	45	18	17.79	0.0307
GO:0008150	biological_process	101	36	39.93	0.0345

GO:0030307	positive regulation of cell growth	8	5	3.16	0.0381
GO:0043066	negative regulation of apoptotic process	33	15	13.05	0.0397

Pathways significantly ($p<0.05$) enriched in proteins significantly associated with RPPA AR at baseline are presented. Enrichment was assessed with Fisher's exact test.

Supplementary Table 6b. Enriched pathways among proteins significantly associated with RRPA AR, evaluated at the change with treatment

GO.ID	Term	Annotated	Significant	Expected	p
GO:0045596	negative regulation of cell differentiation	15	11	8.26	0.0022
GO:0010628	positive regulation of gene expression	38	22	20.91	0.0027
GO:0065003	protein-containing complex assembly	24	15	13.21	0.0054
GO:0050679	positive regulation of epithelial cell proliferation	12	7	6.6	0.0083
GO:0000122	negative regulation of transcription by RNA polymerase II	16	11	8.81	0.0099
GO:0043085	positive regulation of catalytic activity	47	25	25.87	0.0112
GO:0006298	mismatch repair	4	4	2.2	0.0209
GO:0048536	spleen development	4	4	2.2	0.0209
GO:0051496	positive regulation of stress fiber assembly	4	4	2.2	0.0209
GO:1903798	regulation of production of miRNAs involved in gene silencing by miRNA	4	4	2.2	0.0209
GO:0003179	heart valve morphogenesis	4	4	2.2	0.0209
GO:0051592	response to calcium ion	4	4	2.2	0.0209
GO:0003018	vascular process in circulatory system	4	4	2.2	0.0209
GO:0001701	in utero embryonic development	13	9	7.16	0.0281
GO:0008150	biological_process	101	50	55.59	0.0345
GO:0048538	thymus development	8	6	4.4	0.0376
GO:0051276	chromosome organization	16	12	8.81	0.0421
GO:0008285	negative regulation of cell population proliferation	22	11	12.11	0.0442
GO:0035556	intracellular signal transduction	81	41	44.58	0.0463
GO:0043604	amide biosynthetic process	10	8	5.5	0.0481

Pathways significantly ($p<0.05$) enriched in proteins significantly associated with RPPA AR when evaluated at the change in treatment from baseline to week5 are presented. Enrichment was assessed with Fisher's exact test.

Supplementary Table 7: RPPA Antibodies

Cat #	Antibody/ Reagent	Company	Array Dilution	Host
9451	4E-BP1 (S65)	CellSig	1:50	R
9455	4E-BP1 (T70)	CellSig	1:200	R
3661	Acetyl-CoA Carboxylase (S79)	CellSig	1:50	R
9271	AKT(S473)	CellSig	1:100	R
9275	AKT (T308)	CellSig	1:100	R
3348	ALK (Y1586)	CellSig	1:200	RmAb
3341	ALK (Y1604)	CellSig	1:50	R
4184	AMPKalpha1 (S485)	CellSig	1:50	R
4188	AMPKalpha (T172)	CellSig	1:2000	RmAb
ab47563	Androgen Rec (S650)	Abcam	1:1000	R
07-1375	Androgen Rec (S81)	Millipore	1:1000	R
3202	Androgen Receptor total	CellSig	1:200	R
31-1109-00	AR-V7	RevMAb Biosciences	1:50	R
3761	ASK1 (S83)	CellSig	1:50	R
9225	ATF-2 (T69/71)	CellSig	1:500	R
5883	ATM (S1981) (D6H9)	CellSig	1:50	RmAb
4331	ATP-Citrate Lyase (S454)	CellSig	1:100	R
2853	ATR (S428)	CellSig	1:50	R
2914	Aurora A (T288)/B (T232)/C (T198)	CellSig	1:50	RmAb
5724	AXL(Y702)	CellSig	1:50	R
9291	BAD (S112)	CellSig	1:200	R
9295	BAD (S136)	CellSig	1:50	R
2827	BCL-2 (S70)	CellSig	1:50	RmAb
2696	B-RAF (S445)	CellSig	1:50	R
9009	BRCA1 (S1524)	CellSig	1:200	R
2864	cABL (T735)	CellSig	1:50	R
2861	cABL (Y245)	CellSig	1:100	R
9661	Caspase-3, cleaved (D175)	CellSig	1:50	R
9501	Caspase-9, cleaved (D330)	CellSig	1:50	R
9561	Catenin, (beta) (S33/37/T41)	CellSig	1:100	R
9565	Catenin, (beta) (T41/S45)	CellSig	1:50	R
ab5690	CD3 epsilon total	Abcam	1:100	R
2341	CHK1 (S345)	CellSig	1:50	R
2665	CHK2 (S33/35)	CellSig	1:50	R
3073	cKIT (Y703)	CellSig	1:50	R
3391	cKIT (Y719)	CellSig	1:100	R
3313	Cofilin (S3)	CellSig	1:500	RmAb
2831	cPLA2 (S505)	CellSig	1:1000	R
9427	c-RAF (S338)	CellSig	1:200	RmAb
9191	CREB (S133)	CellSig	1:100	R

4656	Cyclin A total	CellSig	1:50	M
4135	Cyclin B1 total	CellSig	1:200	M
554180	Cyclin D1 total	BD	1:100	M
2232	EGFR	CellSig	1:100	R
2234	EGFR (Y1068)	CellSig	1:50	R
44-792	EGFR (Y1148)	Thermo Fisher	1:100	R
44-794	EGFR (Y1173)	Thermo Fisher	1:100	R
2235	EGFR (Y992)	CellSig	1:50	R
9741	eIF4E (S209)	CellSig	1:50	R
2441	eIF4G (S1108)	CellSig	1:1000	R
9181	ELK1 (S383)	CellSig	1:100	R
9575	eNOS (S113)	CellSig	1:50	R
9571	eNOS (S1177)	CellSig	1:50	R
07-357	eNOS/NOS III (S116)	Millipore/Sigma	1:500	R
2242	ErbB2/HER2 total	CellSig	1:100	R
IMG-90189	ErbB2/HER2 (Y1248)	Imgenex	1:500	R
IMG-90185	ErbB2/HER2 (Y877)	Imgenex	1:500	R
4754	ErbB3/HER3 (1B2)	CellSig	1:500	RmAb
4791	ErbB3/HER3 (Y1289)	CellSig	1:200	R
4795	ERBB4 total	CellSig	1:50	RmAb
4757	ErbB4/HER4 (Y1284)	CellSig	1:100	R
9101	ERK 1/2 (T202/Y204)	CellSig	1:1000	R
2511	Estrogen Rec a (S118)	CellSig	1:1000	M
M7047	Estrogen Rec alpha total	DAKO	1:50	M
2781	FADD (S194)	CellSig	1:100	R
3281	FAK (Y576/577)	CellSig	1:200	R
3471	FGF Receptor (Y653/654)	CellSig	1:1000	R
9464	FOXO1 (T24)/FOXO3a (T32)	CellSig	1:200	R
06-953	FOX03a (S253)	Upstate	1:1000	R
9461	FOXO1 (S256)	CellSig	1:100	R
14655	FOXM1 (T600)	CellSig	1:100	RmAb
3231	GAB1 (Y627)	CellSig	1:1000	R
9331	GSK-3alpha/beta (S21/S9)	CellSig	1:100	R
9718	H2A.X S139	CellSig	1:50	R
2573	Heregulin total	CellSig	1:100	R
610958	HIF-1alpha	BD	1:50	M
06-570	Histone H3 (S10) Mitosis Marker	Millipore/Sigma	1:200	R
sc-53319	HLA-DR total	SantaCruz	1:50	M
sc-53302	HLA-DR/DP/DQ/DX total	SantaCruz	1:50	M
3488	HSP90a (T5/7)	CellSig	1:100	R
3021	IGF-1 Rec (Y1131)/Insulin Rec (Y1146)	CellSig	1:500	R
3024	IGF-1R (Y1135/36)/IR (Y1150/51)	CellSig	1:500	RmAb
9246	IkappaB-alpha (S32/36)	CellSig	1:100	M
3025	Insulin Receptor beta total	CellSig	1:200	RmAb

2386	IRS-1 (S612)	CellSig	1:200	R
2382	IRS1 total	CellSig	1:500	R
3331	JAK1 (Y1022/1023)	CellSig	1:50	R
4406	JAK2 (Y1007)	CellSig	1:200	RmAb
M7240	Ki67 (MIB-1)	DAKO	1:100	M
2775	LC3B total	CellSig	1:100	R
3055	LKB1 (S334)	CellSig	1:50	R
3155	M-CSF Receptor (Y723)	CellSig	1:100	RmAb
3521	MDM2 (S166)	CellSig	1:100	R
9121	MEK1/2 (S217/S221)	CellSig	1:500	R
3126	MET (Y1234/Y1235)	CellSig	1:200	R
3515	MLH1 total	CellSig	1:500	M
2017	MSH2 total	CellSig	1:2000	RmAb
3996	MSH6 total	CellSig	1:100	R
9594	MSK1 (S360)	CellSig	1:50	R
2971	mTOR (S2448)	CellSig	1:100	R
3031	NF-kappaB p65 (S536)	CellSig	1:100	R
ab62352	NRF2 total	Abcam	1:200	R
71-7700	p27 (T187)	Invitrogen	1:200	R
9211	p38 MAP Kinase (T180/Y182)	CellSig	1:100	R
9282	p53 total	CellSig	1:5000	R
9284	p53 (S15)	CellSig	1:1000	R
9208	p70 S6 Kinase (S371)	CellSig	1:50	R
9205	p70 S6 Kinase (T389)	CellSig	1:100	R
07-018	p70 S6 Kinase (T412)	Millipore/Sigma	1:500	R
9341	p90RSK (S380)	CellSig	1:200	R
9344	p90RSK (T359/S363)	CellSig	1:200	R
2605	PAK1 (S199/S204)/PAK2 (S192/S197)	CellSig	1:50	R
2601	PAK1 (T423)/PAK2 (T402)	CellSig	1:100	R
9541	PARP, cleaved (D214)	CellSig	1:100	R
2992	PDGF Receptor alpha (Y754)	CellSig	1:500	RmAb
07-021	PDGF Receptor beta (Y716)	Millipore/Sigma	1:200	R
3161	PDGF Receptor beta (Y751)	CellSig	1:50	R
3061	PDK1 (S241)	CellSig	1:200	R
M3653	PD-L1 (22C3)	DAKO	1:50	M
4228	PI3K p85 (Y458)/p55 (Y199)	CellSig	1:100	R
4781	PKA C (T197)	CellSig	1:200	R
06-822	PKC alpha (S657)	Millipore/Sigma	1:1000	R
2821	PLCgamma1 (Y783)	CellSig	1:100	R
2039	PP2A alpha subunit total	CellSig	1:1000	R
4953	PP2A beta subunit total	CellSig	1:1000	R
44-1100	PRAS40 (T246)	Millipore/Sigma	1:1000	R
2611	PRK1 (T774)/PRK2 (T816)	CellSig	1:100	R
3171	Progesterone Receptor (S190)	CellSig	1:50	R

9552	PTEN total	CellSig	1:50	R
9551	PTEN (S380)	CellSig	1:500	R
9421	Raf (S259)	CellSig	1:100	R
3321	Ras-GRF1 (S916)	CellSig	1:50	R
3590	Rb (S780)	CellSig	1:2000	R
3221	Ret (Y905)	CellSig	1:100	R
5176-1	Ron (Y1353)	Epitomics	1:1000	RmAb
9348	RSK3 (T356/S360)	CellSig	1:500	R
4856	S6 Ribosomal Protein (S235/236)	CellSig	1:200	R
2215	S6 Ribosomal Protein (S240/244)	CellSig	1:1000	R
9251	SAPK/JNK (T183/Y185)	CellSig	1:100	R
5599	SGK1 (S78)	CellSig	1:100	R
07-206	Shc (Y317)	Millipore/Sigma	1:200	R
3104	Smad2 (S245/250/255)	CellSig	1:100	R
2105	Src (Y527)	CellSig	1:200	R
2101	Src Family (Y416)	CellSig	1:200	R
07-307	Stat1 (Y701)	Millipore/Sigma	1:500	R
4441	Stat2 (Y690)	CellSig	1:100	R
9134	Stat3 (S727)	CellSig	1:100	R
9145	Stat3 (Y705)	CellSig	1:100	RmAb
5267	Stat4 (Y693)	CellSig	1:100	R
9351	Stat5 (Y694)	CellSig	1:50	R
9361	Stat6 (Y641)	CellSig	1:100	R
90540	TROP2	CellSig	1:750	R
3614	Tuberin/TSC2 (Y1571)	CellSig	1:50	R
9321	Tyk2 (Y1054/1055)	CellSig	1:500	R
2478	VEGFR 2 (Y1175)	CellSig	1:50	RmAb
2471	VEGFR 2 (Y951)	CellSig	1:50	R
2474	VEGFR 2 (Y996)	CellSig	1:100	R
13008	YAP (S127)	CellSig	1:100	RmAb
